# Catalysis of a 1,3-dipolar reaction by distorted DNA incorporating a heterobimetallic platinum(ii) and copper(ii) complex†
†Electronic supplementary information (ESI) available: Experimental details, characterization of cycloadducts and intermediates, computational data. CCDC 1411372 and 1411373. For ESI and crystallographic data in CIF or other electronic format see DOI: 10.1039/c7sc02311a



**DOI:** 10.1039/c7sc02311a

**Published:** 2017-08-22

**Authors:** Iván Rivilla, Abel de Cózar, Thomas Schäfer, Frank J. Hernandez, Alexander M. Bittner, Aitziber Eleta-Lopez, Ali Aboudzadeh, José I. Santos, José I. Miranda, Fernando P. Cossío

**Affiliations:** a Department of Organic Chemistry I , Centro de Innovación en Química Avanzada (ORFEO-CINQA) , Universidad del País Vasco/Euskal Herriko Unibertsitatea (UPV/EHU) , Donostia International Physics Center (DIPC) , Po Manuel Lardizabal 3 , E-20018 Donostia/San Sebastián , Spain . Email: fp.cossio@ehu.es; b Ikerbasque, Basque Foundation for Science , Ma Díaz de Haro 3 , E-48013 Bilbao , Spain; c NanoBioSeparations Group , POLYMAT University of the Basque Country (UPV/EHU) , Avda. Tolosa 72 , E-20018 Donostia/San Sebastián , Spain; d CIC NanoGUNE , Consolider. Tolosa Hiribidea, 76 , E-200018 Donostia/San Sebastián , Spain; e SGIker NMR Facility , Universidad del País Vasco/Euskal Herriko Unibertsitatea (UPV/EHU) , Avda. Tolosa 72 , E-20018 Donostia/San Sebastián , Spain

## Abstract

A novel catalytic system based on covalently modified DNA is described. This catalyst promotes 1,3-dipolar reactions between azomethine ylides and maleimides. The catalytic system is based on the distortion of the double helix of DNA by means of the formation of Pt(ii) adducts with guanine units. This distortion, similar to that generated in the interaction of DNA with platinum chemotherapeutic drugs, generates active sites that can accommodate *N*-metallated azomethine ylides. The proposed reaction mechanism, based on QM(DFT)/MM calculations, is compatible with thermally allowed concerted (but asynchronous) [π4s + π2s] mechanisms leading to the exclusive formation of racemic *endo*-cycloadducts.

## Abstract 2

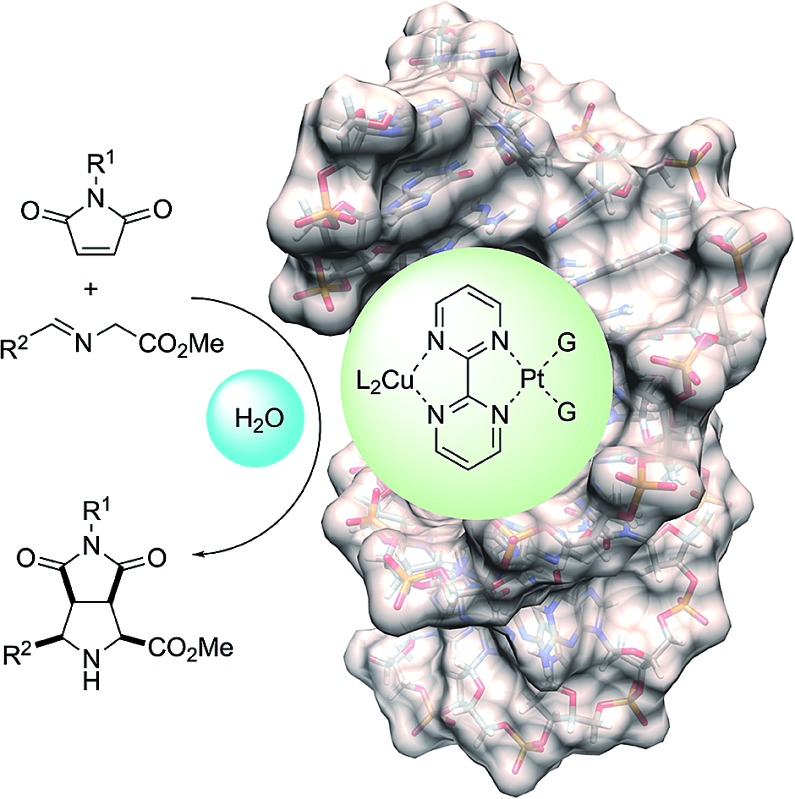
A novel catalytic system based on covalently modified DNA is described.

## Introduction

The main role of DNA is the storage of genomic information leading to the biosynthesis of proteins *via* diverse forms of RNA.^1^ In turn, proteins play multiple roles in living systems, catalysis being among the most important ones. This general pattern has been refined as a consequence of the discovery of catalytic RNAs (ribozymes)^2^ and DNAs (deoxyribozymes).^3^ However, since DNA is structurally less versatile than RNA and proteins, additional molecules and functional groups are required to expand the catalytic space of DNA. Roelfes and Feringa^4^ brilliantly demonstrated the feasibility of this concept by intercalation of aza-chalcones in the double helix and subsequent coordination to copper salts. Further work^5^ demonstrated that various intercalating heterocycles and metallic salts in the presence of DNA are able to promote, among other transformations, Diels–Alder reactions,^4,6^ Friedel–Crafts alkylations^7^ and Michael^8,9^ (including oxa-Michael)^10,11^ additions. It is remarkable that all these reactions were carried out in water, although in several cases the system tolerated organic co-solvents.^7^ Other DNA activation methods include covalent attachment of active catalytic sites such as proline organocatalysts,^12^ as well as Ir(i)^13^ and Pt(ii)^14^ complexes.

Despite the relevance of (3 + 2) cycloadditions in the chemical synthesis of five-membered rings,^15^ the enzymatic version of this reaction has not been identified in living systems.^16,17^ Only a very recent example of a possible enzymatic 1,3-dipolar reaction has been reported to date.^18^ In addition, nonenzymatic 1,3-dipolar reactions have been postulated in the biosynthesis of several alkaloids^19^ and natural products such as furanocembranoids^20,21^ and santolin Y.^22^ Therefore, to the best of our knowledge a biomolecule-assisted *bona fide* (3 + 2) cycloaddition between azomethine ylides and alkenes to produce unnatural proline derivatives has not been reported to date. Within this context, we herein describe the first example of a DNA-assisted 1,3-dipolar reaction in water.

## Results and discussion

**Scheme 1:**
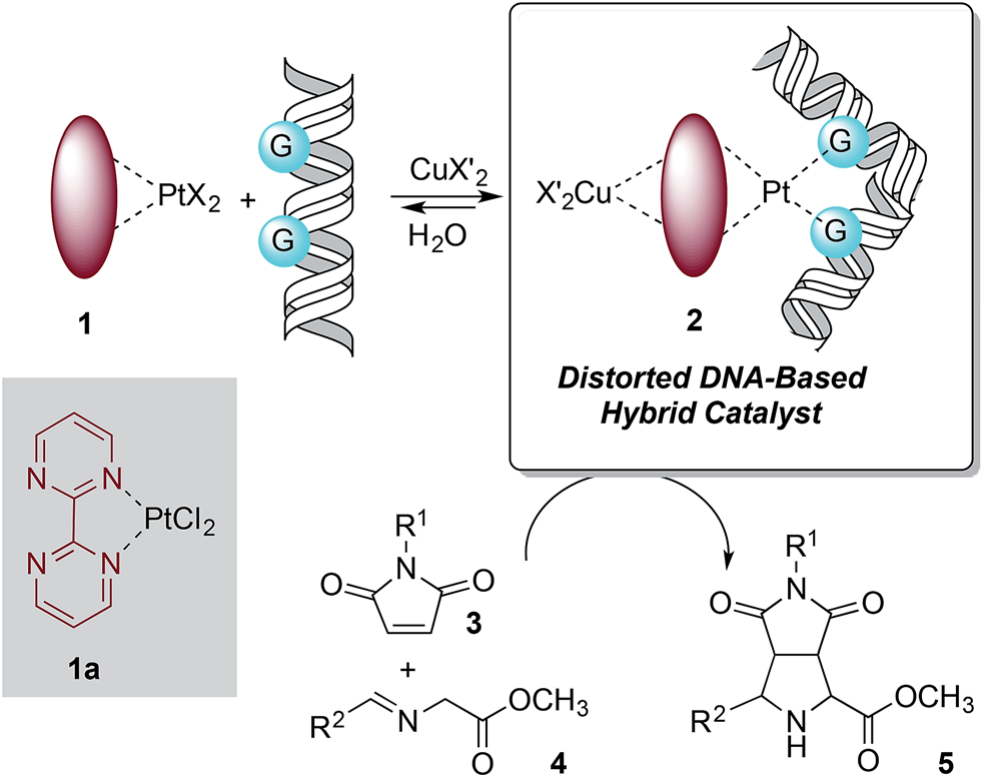
Basic design of DNA-based catalysts for 1,3-dipolar reactions. The distortion of DNA by coordination of G bases with Pt(ii) is shown.

The design of the covalent modification of DNA was based on the ability of Pt(ii) chemotherapeutic drugs to bind mainly 1,2-intrastrand GpG units,^23,24^ thus providing a concave-convex distortion of the double helix that could mimic the active sites of metalloenzymes. In previous work^25^ we reported new chiral ligands that can bind Cu(ii) salts^26^ and efficiently catalyze (3 + 2) cycloadditions involving azomethine ylides. Therefore, we reasoned that a DNA–Pt(ii)–Cu(ii) heterobimetallic complex similar to that depicted in Scheme 1 could catalyze this reaction.

**Fig. 1:**
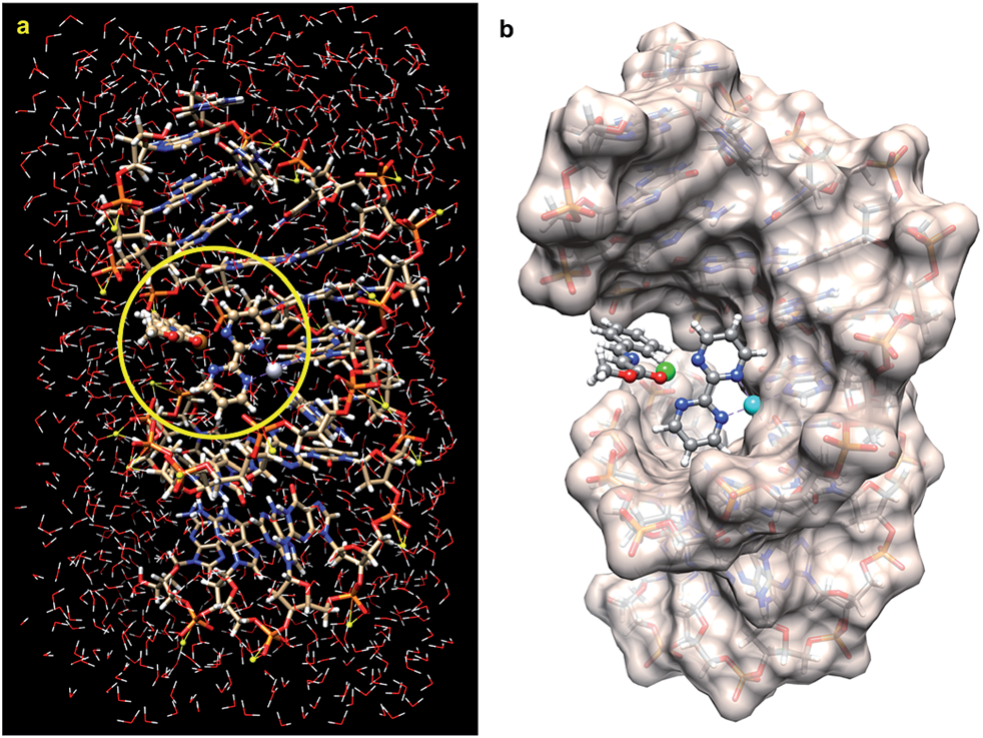
(a) Fully optimized (B3LYP/LanL2DZ:UFF level of theory) structure of a DNA double strand containing the d(CCTCTG*G*TCTCC)-d(GGAGACCAGAGG) sequence, in which the complex **1a** is bound to the G*pG* unit and to the N–Cu(ii) azomethine ylide derived from imine **4a** (R^2^ = Ph, Scheme 1), surrounded by 509 water molecules and 22 sodium cations. The QM part of the optimization is within the circle highlighted in yellow. (b) The same optimized structure but showing the solvent accessible surface of the DNA fragment. The water molecules have been removed for clarity. Pt(ii) and Cu(ii) atoms are represented in light blue and green, respectively.

First, we tested the feasibility of this basic design using computational methods. As starting point we took the crystal structure reported by Takahara *et al.*^27^ for a cisplatin-double-stranded oligodeoxynucleotide complex (pdb code: ; 1aio), where G*G* denotes a 1,2-intrastrand *cis*-[(H3N)2Pt-d(GpG)] crosslink. To this structure 2,2′-bipyrimidin (bipym) was added and the azomethine ylide derived from methyl (*E*)-2-(benzylidene-amino)acetate **4a** was coordinated to a copper(ii) metallic centre. The resulting structure was stabilized by incorporating 509 water molecules and 22 sodium cations (Fig. 1a). The whole ensemble was optimized using a hybrid QM/MM ONIOM^28–30^ scheme (ESI†). The full structure thus optimized was found to keep the folded geometry of the distorted double helix, where the Cu(ii)–bipym–Pt(ii)–G*G* system generated a cavity to which the azomethine ylide was coordinated.

**Fig. 2:**
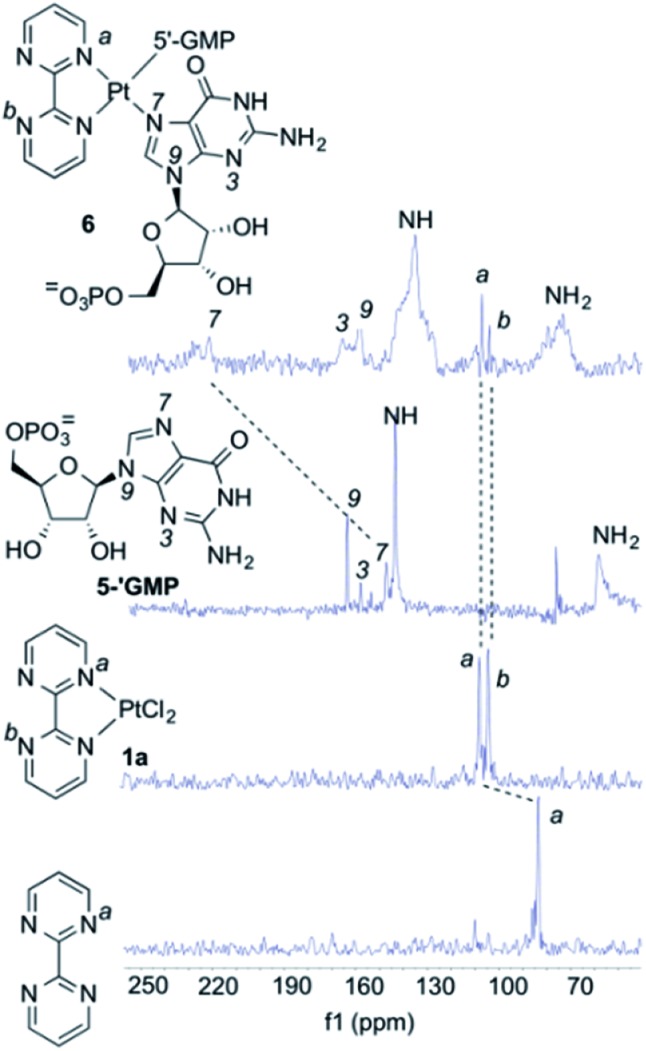
CP-MAS experiments with (bipym)PtCl_2_ (**1a**) and 5′-GMP. The spectra of 2,2′-bipyrimidine, **1a**, 5′-GMP and the complex **6** resulting from the combination between **1a** and two equivalents of 5′-GMP are displayed. All spectra correspond to ^15^N-NMR scans obtained by ^5^N–^1^H cross-polarization experiments in the solid state. The assignments of the ^15^N *δ*-values for the different nitrogen atoms are shown. The most significant changes in *δ*-values upon coordination to Pt(ii) are highlighted.

As it can be seen by inspection of Fig. 1b, the resulting ensemble closely resembles the active site of a metalloenzyme. As a proof-of-concept experiment, we next examined the ability of (bipym)PtCl_2_ complex **1a** to bind two equivalents of guanosine. Since **1a** and its derivatives posed solubility problems, we monitored the different species in the solid state using Cross Polarization-Magic Angle Spinning (CP-MAS) spectroscopy.^31^ The NMR spectrum of bipym showed only one ^15^N-NMR signal, as expected from its *D*_2h_ symmetry (Fig. 2). In contrast, the two nitrogen atoms of **1a** coordinated to Pt(ii) could be readily distinguished. Then, we used 5′-GMP as a suitable equivalent of G units in DNA and analysed the ^1^H–^15^N CP-MAS spectra at different mixing times to assign the five nitrogen atoms of the G-unit. Combination of **1a** and 5′-GMP resulted in the formation of an adduct whose ^15^N chemical shift associated with the N7 atom of the purine system was considerably deshielded with respect to the signal recorded for 5′-GMP (Fig. 2).

We concluded that Pt(ii) complex interacts with guanine units yielding a square planar complex, in which two equivalent G units bind the Pt(ii) centre by means of the respective N7 atoms of the purine bicycle. We next studied the interaction between **1a** and oligodeoxynucleotides in order to assess the binding abilities of different DNA sequences.

**Fig. 3:**
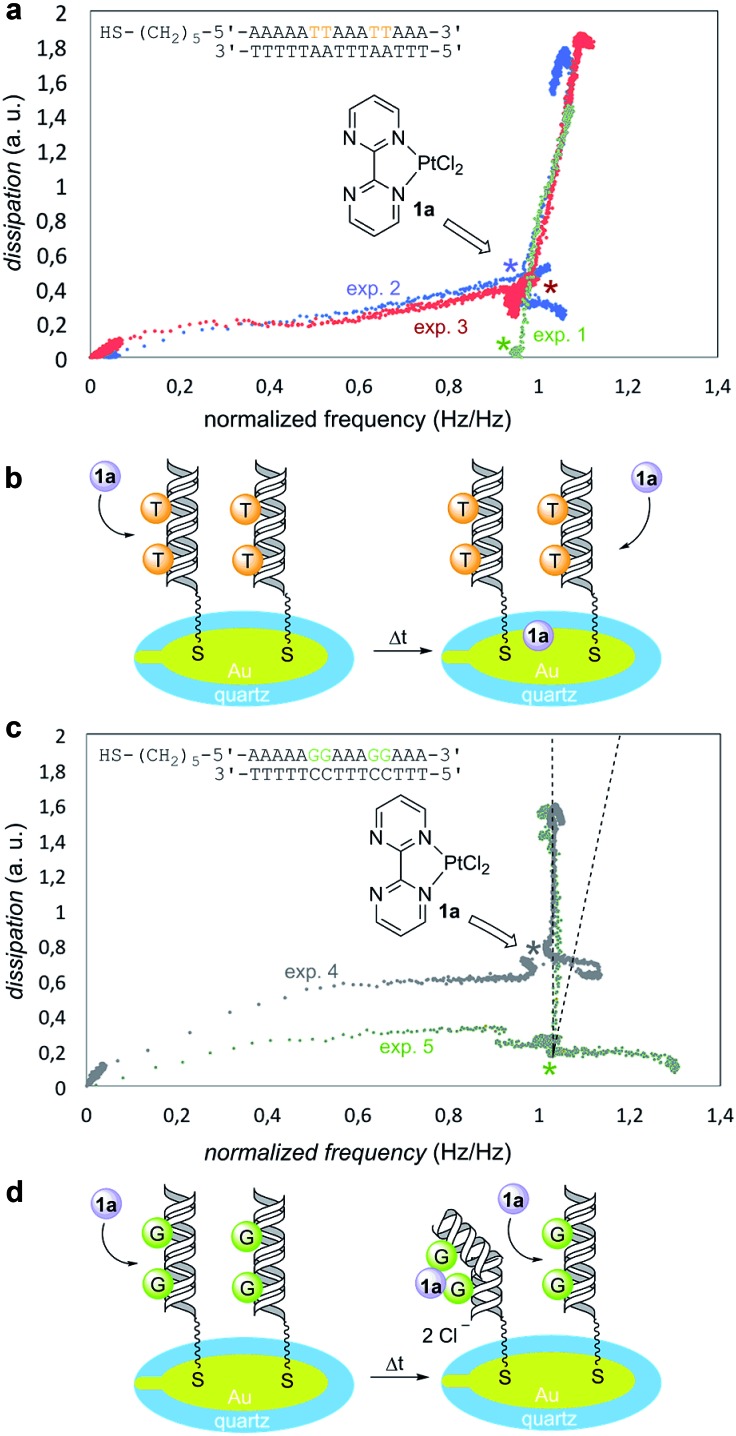
(a) Quartz-crystal microbalance with dissipation (QCM-D experiments) monitoring of non-binding substrate (duplicate experiments 2 and 3). Experiment 1 data (in green) correspond to the blank experiment. Dissipation *vs.* normalized frequency plots at different times are displayed. Starting times correspond to the bottom-left corner of graph. (b) Cartoon representing the behavior of a non-binding oligodeoxynucleotide. (c) QCM-D scans showing the behavior of a GpG-containing oligodeoxynucleotide (duplicate experiments 4 and 5). (d) Cartoon representing specific binding between **1a** and immobilized oligodeoxynucleotides.

To this end, we used a quartz-crystal microbalance with dissipation monitoring (QCM-D) device.^32^ These QCM-D experiments measured changes in frequency, which correlate with mass adsorption on the sensor surface, and the dissipation of energy of the adsorbed film, which in turn correlates with its viscoelasticity.^33,34^ The dissipation *vs.* frequency points were collected for various times and ordered graphically from left to right and from bottom to top (Fig. 3). In a reference experiment denoted as “exp. 1” in Fig. 3a, the Pt(ii) complex **1a** was injected on bare gold (no DNA immobilized) in PBS buffer. Under these conditions, dissipation increased linearly with frequency, thus resulting in an incremental dissipation entirely correlated with the mass adsorbed on the surface. To carry out the experiments in the presence of DNA, we selected two different model oligodeoxynucleotides on the basis of the well-known binding ability of GpG pairs with platinum drugs.^23,24^ We tested in duplicate the oligomer containing 5′-thiol-AAAAATTAAATTAAA-3′ binding sequence (Fig. 3a, experiments 2 and 3). An overlay with the reference experiment 1 revealed that this response was indeed non-specific as both experiments showed practically identical profiles with respect to the reference. We interpreted these results as negative experiments in which there was no significant interaction between Pt(ii) complex **1a** and G-free deoxyoligonucleotide (Fig. 3b). We next examined the behavior of the oligomer containing 5′-thiol-AAAAA**GG**AAA**GG**AAA-3′ binding sequence (Fig. 3c, experiments 4 and 5). In this case, upon addition of **1a** both plots showed an identical increase in dissipation while simultaneously no frequency change was observed, in sharp contrast with the previous blank and negative experiments 1–3. This indicated that first, there was no measurable non-specific binding occurring as it would be detected as a change in frequency (see the dotted lines in Fig. 3c); second, injection of **1a** induced a strong change in dissipation, which can be only attributed to conformational changes taking place in the GpG pairs-containing immobilized and hybridized deoxyoligo-nucleotide. From these results, we concluded that GpG-containing DNA interacts specifically with Pt(ii) complex **1a** resulting in an increasingly more dissipative film, which in turn correlates with additive conformational changes of DNA upon addition of **1a** (Fig. 3d).

**Fig. 4:**
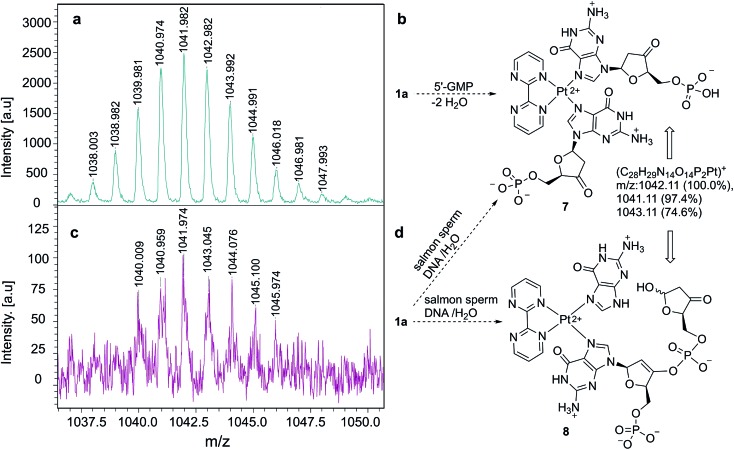
(a) High *m*/*z* region of MALDI-TOF mass spectrum corresponding to the complex formed between **1a** and 5′-GMP. (b) Structure of ion **7**, associated with the spectrum shown in (a). (c) High *m*/*z* region of MALDI-TOF mass spectrum corresponding to the complex formed between **1a** and salmon sperm-DNA. (d) Structures of ions **7** and **8**, associated with the spectrum shown in (c), the latter corresponding to a complex formed from two consecutive G units.

Once we verified that complex **1a** binds selectively G-containing oligonucleotides, we tested its binding ability to DNA strands. As a control experiment, we mixed **1a** with 5′-GMP and the resulting complex was analyzed by MALDI-TOF mass spectrometry.^35,36^ We observed an ensemble of high mass-to-charge (*m*/*z*) signals distributed around a value of *m*/*z* ≈ 1042 a.u., as shown in Fig. 4a. These signals correlate satisfactorily with complex **7** (Fig. 4b), closely related to **6** (Fig. 2), in which two ketone units have been generated *via* dehydration-tautomerization of one hydroxy group of each ribose unit. After this control experiment, the same protocol was followed, but instead of 5′-GMP we used DNA sodium salt from salmon sperm (salmon sperm DNA in Fig. 4) with a % G-C content of 41.2% and a molecular mass of 1.3 × 10^6^ Da (*ca.* 2000 bp). In this case, the same profile was observed in the corresponding MALDI-TOF mass spectrum (Fig. 4c), with a high *m*/*z* ensemble centered at *ca.* 1042 a.u. This response was interpreted in terms of ion **7** or, for two consecutive GpG units in the starting salmon sperm DNA, as the keto-enol phosphoric ester depicted as ion **8** in Fig. 4d. On the basis of these experiments, we concluded that the **1a**–G_2_ complexes observed in monomeric and oligomeric G-containing species can be extended to double strain DNA.

**Fig. 5:**
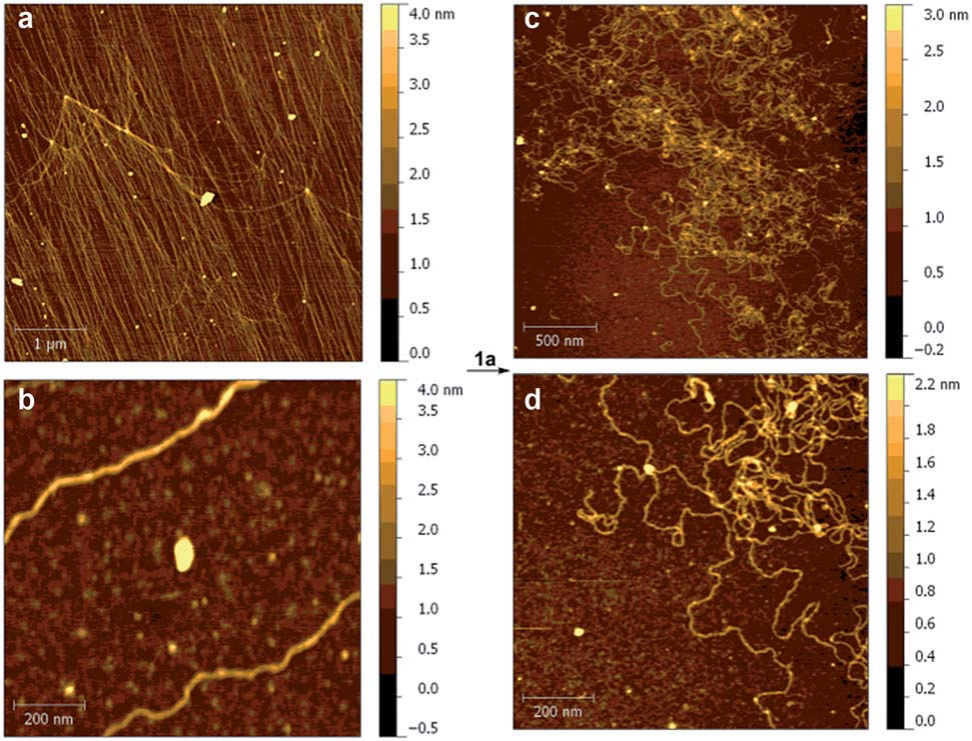
Atomic force microscopy (AFM) analysis of the interaction between DNA and **1a**. AFM scans of λ-DNA before (a, b) and after (c, d) incubation with **1a** in solution to yield intra- and interstrand *cis*-{(bipym)Pt(d[GpG + GpXpG + GpA])} adducts. The colour code represents the height (in nm) with respect to the bare surface.

We also studied the effect of Pt(ii) complex **1a** on the structure of DNA strands. Thus, samples of DNA (*ca.* 48 kb), from λ phage-infected *E. coli* were analyzed by atomic force microscopy (AFM).^37^ The corresponding DNA strands were unambiguously identified on oxidized silicon^38^ by the corresponding AFM images (Fig. 5a and b). When **1a** was added, the AFM scans showed large morphological changes consisting of local kinks and crosslinks (Fig. 5c and d). On the basis of the previously presented experiments, these changes were attributed to the formation of intra- and interstrand *cis*-{(bipym)Pt(d[GpG + GpXpG + GpA])} adducts.^39^

These advanced analytical studies permitted us to establish the main features of the Pt(ii)-mediated binding between molecule **1a** and DNA. We concluded that the thus generated hybrid system could catalyse 1,3-dipolar reactions provided that (i) the heterobimetallic Cu(ii)–Pt(ii) centre is able to generate *in situ* the required *N*-metallated azomethine ylide derived from the corresponding imine **4**; and (ii) the active site can accommodate the dipolarophile **3**.

**Scheme 2:**
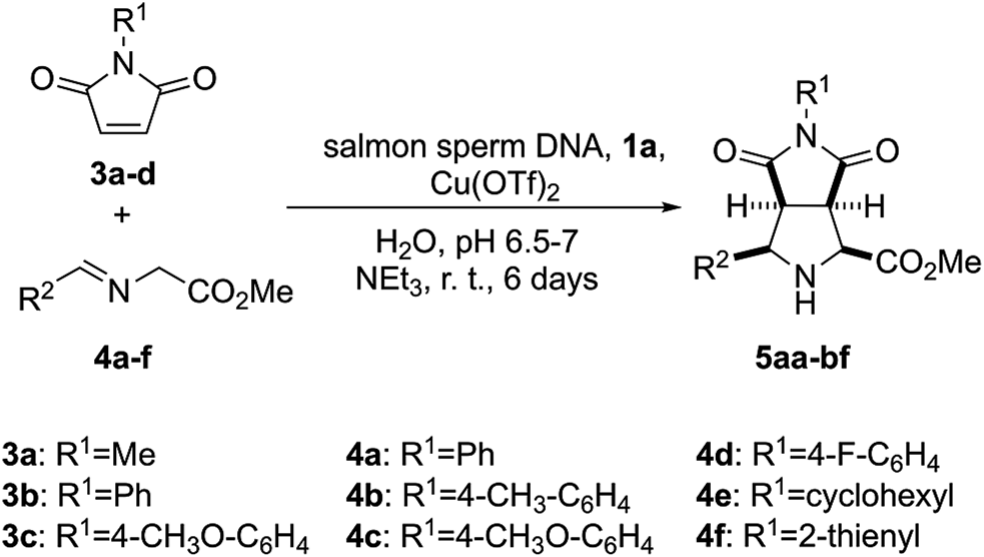
Synthesis of (3 + 2) *endo*-cycloadducts **5aa–5bf** catalysed by the heterobimetallic complex formed by salmon sperm DNA and **1a** in the presence of copper(ii) triflate.

In order to test the catalytic ability in (3 + 2) cycloadditions of the DNA–heterobimetallic complex, we prepared catalyst **2** (Scheme 1) by using salmon sperm DNA in a buffered solution of (*N*-morpholino)propane-sulfonic acid (MOPS), to which **1a** was added, followed by copper(ii) triflate, triethylamine and the corresponding maleimide **3** and imine **4** (Scheme 2). After six days of reaction at room temperature, nine distinct *endo*-(3 + 2)-cycloadducts (**5aa–5bf**, Table 1) could be generated. This *endo* stereochemistry was secured on the basis of the spectroscopic data and, in two cases, by X-ray diffraction analysis (ESI†).

**Table 1:** Yields observed in the formation of bicyclic cycloadducts **5aa–5bf** from (3 + 2) cycloadditions between maleimides **3a–c** and imines **4a–f** catalysed by an heterobimetallic complex formed by low weight salmon sperm DNA, **1a** and Cu(OTf)_2_

Entry	R^1^ (**3**)	R^2^ (**4**)	**5**	Yield^*a*^ (mmol × 10^–3^) [%]
1	Me (**3a**)	Ph (**4a**)	**5aa**	10.5 ± 0.4 [21]
2	Ph (**3b**)	Ph (**4a**)	**5ba**	18.0 ± 0.5 [36]
3	4-MeO–C_6_H_4_ (**3c**)	Ph (**4a**)	**5ca**	10.0 ± 0.8 [20]
4	Ph (**3b**)	4-Me–C_6_H_4_ (**4b**)	**5bb**	17.9 ± 0.4 [23]
5	Ph (**3b**)	4-MeO–C_6_H_4_ (**4c**)	**5bc**	17.5 ± 0.3 [35]
6	Ph (**3b**)	4-F-C_6_H_4_ (**4d**)	**5bd**	12.5 ± 0.2 [25]
7	Ph (**3b**)	Cyclohexyl (**4e**)	**5be**	13.5 ± 0.4 [27]
8^*b*^	Ph (**3b**)	2-Thyenyl (**4f**)	**5bf**	16.0 ± 0.9 [32]

^*a*^Yields of isolated pure products correspond to averaged values obtained after three experiments. The limiting reactant was the corresponding maleimide **3** (0.05 mmol).

^*b*^Structure confirmed by X-ray diffraction analysis (ESI).^40^

In order to assess the relevance of each component of the catalytic system we tested 17 possibilities resulting from the combination of all the reagents except at least one (see Table S1 of the ESI†). In all these control experiments no 1,3-dipolar reaction was observed. In particular, when different combinations of triethylamine and the Cu(ii) and Pt(ii) salts were tested in the absence of salmon sperm DNA, the reaction did not proceed. Similarly, different combinations in the presence of salmon sperm DNA but in the absence of base, 2,2′-bipyrimidine and/or one of the metals did not produce any (3 + 2) cycloadduct. It is interesting to note that the combination of DNA, 2,2′-bipyrimidine and Cu(OTf)_2_ in the absence of Pt(ii) was also unproductive (see Table S1 of the ESI,† entry 13), thus indicating that the powerful Roelfes–Feringa method consisting of a metallated intercalating heterocycle cannot catalyse this challenging 1,3-dipolar cycloaddition. In addition, these experiments demonstrate that there is no background reaction (see in particular entry 1 of Table S1 of the ESI†). In summary, these control experiments demonstrated that combination of 2,2′-bipyrimidine, Pt(ii), Cu(ii) and DNA, most likely by bonding to consecutive GG units, is required to achieve moderate yields of (3 + 2) racemic *endo*-cycloadducts **5** (see Table S1 of the ESI,† entry 10).

We interpreted our results as follows: Previously formed DNA-**1a** adducts bound Cu(OTf)_2_ and the resulting heterobimetallic complex **2a** (Fig. 5a) coordinated imine **4** to form intermediate species **INT1**, from which the corresponding *N*-metallated azomethine ylide **INT2** was formed *via* triethylamine-assisted deprotonation. This 1,3-dipole interacted with dipolarophile **3** to form the corresponding (3 + 2) cycloadduct and regenerating **INT1***via* interaction with another equivalent of imine, thus completing the catalytic cycle. In order to understand the origins of the *endo* selectivity we optimized the possible *endo*- and *exo*-transition structures under the same computational framework used to optimize the structure of **INT2** (Fig. 5b and c). Both saddle points were found to be quite asynchronous but still associated with a concerted [π4s + π2s] symmetry allowed mechanism, as indicated by the bond distances corresponding to the formation of the two new σ-bonds. We also found that *endo*-**TS** was stabilized by a strong electrostatic interaction between the nitrogen atom of the imide moiety and the metallic centre (Fig. 5b). As a result, *exo*-**TS** was calculated to be *ca.* 13 kcal mol^–1^ higher in energy than its *endo* congener, thus predicting the preferential formation of cycloadduct *endo*-**5aa** under kinetic control, in nice agreement with the experimental data.

**Fig. 6:**
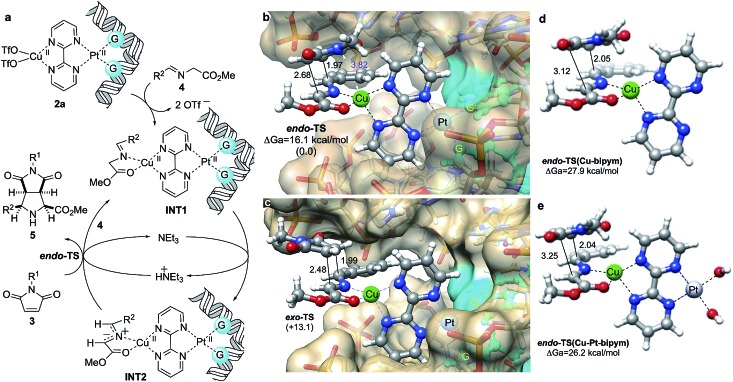
(a) Proposed catalytic cycle for (3 + 2) cycloadditions between azomethine ylides **INT1** and maleimides **3** catalyzed by *in situ* formed DNA–bipym–Pt(ii)–Cu(ii) complexes. (b, c) Fully optimized geometries and relative energies of *endo*- and *exo*- transition structures (TS) associated with the **3a** + **4a** → **5aa** reaction. Numbers in parentheses are the relative energies, in kcal mol^–1^. Bond distances are given in Å. Guanine units bound to Pt(ii) are highlighted in blue. Calculations were performed at the B3LYP/LanL2DZ:UFF level of theory as indicated in Fig. 1b. Water molecules and sodium cations were included in the calculations (MM level, UFF force field) but are not shown for clarity. (d, e) Same transition structures associated with the formation of cycloadduct *endo*-**5aa** in the absence of DNA and Pt(ii) (d), and in the absence of DNA (e). In the latter case, two binding water ligands have been included. Also in (d) and (e), water molecules were included in the calculations (MM level, UFF force field, not shown for clarity).

We performed similar calculations in the absence of DNA. In these studies, the features of the computational model (including surrounding water molecules, see the ESI†) remained identical. The results are gathered in Fig. 6d and e. We observed that in the presence of Cu(ii) and bipyrimidine, the chief features of the transition structure leading to *endo*-**5aa** are similar to those found for the **3a** + **4a** → **5aa** reaction in the presence of DNA. However, the activation energy was found to be *ca.* 12 kcal mol^–1^ higher, which corresponds to a *k*(DNA–Cu–Pt)/*k*(Cu) ratio of *ca.* 4.8 × 10^8^ (Fig. 6d). When the square planar diaqua–Pt(ii) moiety was incorporated to the reaction coordinate (Fig. 6e), the shape of the corresponding saddle point did not change significantly and the activation energy was slightly lower than in the previous case, with a calculated *k*(DNA–Cu–Pt)/*k*(Cu–Pt) ratio of *ca.* 2.7 × 10^7^. It is interesting to note that the DNA-free simulations also predict the preferential formation of the *endo*-cycloadduct (see Fig. S3 and Table S3 of the ESI†). These results are in agreement with the absence of reactivity observed when DNA was not present. We interpret the lower activation energies in the presence of DNA in terms of the destabilization of the substrate by restriction of the conformational freedom and by the electrostatic repulsion between the anionic polyphosphate environment and the anionic part of the starting 1,3-dipole, which is alleviated along the reaction coordinate leading to the non-zwitterionic cycloadduct.

## Conclusions

In this work we have shown that (2,2′-bipym)PtCl_2_ complex **1a** binds 5′-GMP by its N7 atom. In addition, oligomers containing GpG pairs can interact selectively with **1a**, in line with the major formation of *cis*-[Pt(ii){GpG}] complexes in platinum drugs. Moreover, **1a** promotes significant structural changes in λ-DNA, resulting in highly distorted microenvironments around the metallic centres, most likely because of the formation of *cis*-[Pt(ii){GpG} + {GpXpG} + {GpA}] adducts. On the basis of these experiments, we conclude that a suitable bimetallic complex based on Pt(ii) can distort the double helix of DNA to generate an active site similar to those found in well-known metalloenzymes, as supported by QM/MM calculations. Finally, we have shown that the system formed by **1a**, salmon sperm DNA and Cu(OTf)_2_ catalyses (3 + 2) cycloadditions between azomethine ylides (formed *in situ* from the corresponding imines) and maleimides. Only the racemic *endo*-cycloadducts are obtained, probably because of competing geometries of the intermediate *N*-metallated azomethine ylides linked to the **1a**–DNA hybrid system. This result demonstrates that modified biomolecules can catalyse chemical reactions in water, for which there is no equivalent in living systems.

## Experimental section

### Computational methods

All the stationary points were fully optimized and characterized (harmonic analysis) within the QM/MM scheme using B3LYP hybrid functional.^29^ LANL2DZ basis set and effective core potential,^24^ as well as UFF force field^25^ were used within the ONIOM^28^ framework as implemented in the Gaussian09 (ref. 30) suite of programs.

### NMR experiments

Liquid NMR spectra were recorded on either a Bruker Avance 500 MHz or 400 MHz spectrometer at standard temperature and pressure, equipped with an *z* gradient BBO probe. The 15N spectra were recorded using an inverse gated sequence at 40.54 MHz, with a time domain of 32k, a spectral width of 16 kHz, an interpulse delay of 5 s and an acquisition time of 1 s. DMSO-d8 was used as internal standard for 1H and MeNO_2_ (376.86 ppm “Bruker scale”) as external reference for 15N NMR spectra. Solid State NMR spectra were recorded on a Bruker 400 AVANCE III WB spectrometer 9.40 T (1H = 400 MHz). 13C CP-MAS and 1H spectra were collected by using a 4 mm CP-MAS probe at a spinning of 10 kHz. These experiments were carried out using the standard pulse sequence at 100.6 MHz, with a time domain of 2 K, a spectral width of 29 kHz, a contact time of 1.5 ms and an interpulse delay of 5 s for 13C spectra. A nominal frequency of 400 MHz was used for 1H, employing the DUMBO pulse sequence, a time domain of 2 K, a spectral width of 20 kHz and an interpulse delay of 5 s. 15N solid state spectra were collected by using a 7 mm MASDVT probe at a spinning of 5 KHz. 15N CP-MAS NMR spectra were recorded using a standard pulse sequence at 40.56 MHz, a time domain of 1k, a spectral width of 96 kHz, a contact time of 2 ms and an interpulse delay of 5 s.

### MALDI-TOF mass spectrometry

Matrix Assisted Laser Desorption Ionization Time of Flight Mass Spectrometry (MALDI-TOF MS) measurements were performed on a Bruker Autoflex Speed system (Bruker, Germany) equipped with a 355 nm NdYAG laser. Spectra were acquired in the positive reflection mode. *trans*-2-[3-(4-*tert*-Butylphenyl)-2-methyl-2-propenylidene] malonitrile (DCTB, Fluka) was used as a matrix. Sodium trifluoroacetate and silver trifluoroacetate (Aldrich) were added as the cationic ionization agents (10 g L^–1^ dissolved in THF). The matrix was also dissolved in THF at a concentration of 20 g L^–1^. All the samples were dissolved in water at a concentration. The matrix and salt were premixed in a 10 : 1 ratio. Approximately 0.5 μL of the sample were hand spotted on the ground steel target plate and 0.5 μL of the matrix/salt obtained mixture were quickly added to the hand spotted samples. For each spectrum 5000 laser shots were accumulated.

### AFM experiments

DNA samples were prepared from lyophilized lambda phage DNA, methylated from *E. coli* host strain W3110 (*M*_w_. 31.5 × 103 kDa, 48 kb, from sigma Sigma-Aldrich). DNA solutions (0.02 mg mL^–1^ in (*N*-morpholino)propanesulfonic acid, MOPS, 10 mM) were prepared and in each experiment 10 μL were deposited on the freshly cleaned silicon oxide surface. Then 10 μL of DNA solution was deposited on a silicon wafer, freshly hydrolyzed with oxygen plasma. Immediately afterwards the solution droplet was softly blown with a nitrogen stream. A saturated solution of compound **1a** in Millipore water (18 Mohm cm, TOC < 5 ppb) was prepared and a droplet of 20 μL was deposited on DNA/silicon oxide sample. The Pt(ii) salt was incubated for 30 minutes and dried with nitrogen stream. DNA surface topographies were imaged with an atomic force microscopy (AFM 5500, Agilent Technologies/Keysight Technologies) in air, in AC mode with an oscillation frequency of 63 kHz. The images were obtained at 512 and 1024 points per lines. All the data were processed with Gwyddion 2.31 program.^41^

### QCM-D experiments

The experiments were carried out using a quartz-crystal microbalance with dissipation monitoring from Biotin Scientific and respective quartz sensors QSX 301 Gold (Biolin Scientific). For each experiment, a new sensor was used. Each experiment started with obtaining a stable baseline by passing PBS–buffer (Sigma Aldrich) at a flow rate of 100 μL min^–1^ through the sensor module (E1. Biolin Scientific) using a peristaltic pump (Ismatec, Reglo Digital). Frequency and dissipation data were recorded using the acquisition software QSoft 401 (Biolin Scientific). The 5th harmonic of the frequency was used for further data processing as this harmonic has previously proven to yield most reliable signals.^34^ Stability was assumed when the average frequency signal was less than 0.05 Hz during five min. Subsequently, each thiol-containing oligomer (Biomers) was injected (5 μM) at the same feed flow velocity for 10 min, followed by rinsing with PBS buffer during additional 10 min in order to wash-off any loosely bound DNA. The complementary strand (5 mM) was then injected at the same flow rate and duration, followed again by injection of PBS buffer. Finally, the Pt(ii) complex **1a** was injected at a concentration of 0.1 mg mL^–1^, followed by rinsing with buffer. For better visualization, in Fig. 3 the frequency data were normalized for the steady-state frequency (*F*_max_) measured when the DNA duplex had formed after injection of the complementary strand and before injection of the Pt(ii) complex. In this way, the effect of the Pt(ii) complex on the dissipation of the DNA was comparable between measurements irrespective of the absolute DNA coverage of the sensor.

### Reagents and catalysis

[Pt(DMSO)_2_Cl_2_] and [(bipym)PtCl_2_] were prepared following the procedure described in the literature.^42^ The α-imino glycinate esters R^2^HC = NCH_2_CO_2_Me **4**, with R^2^ = Ph (**4a**), *p*-Me(C_6_H_4_) (**4b**), cyclohexyl (**4e**), 3′-thienyl (**4f**), *p*-F(C_6_H_4_) (**4d**), and *p*-OMe(C_6_H_5_) (**4c**), were prepared using the synthetic procedures described in the literature.^25^

#### Synthesis of *endo*-methyl 4,6-dioxo-3-aryloctahydro-pyrrolo[3,4-*c*]pyrrole-1-carboxylates (**5**)

Low molecular weight (*ca.* 200 bp) DNA from salmon sperm (Sigma-Aldrich) was used as purchased. All cycloaddition experiments were carried out in an orbital stirrer at room temperature. In a typical reaction procedure, salmon sperm DNA (1 mmol) was added to 20 mL of a buffered solution of MOPS (20 mM, pH 6.5) and the resulting mixture was stirred for 24 h. Then, **1a** (16 mg, 0.015 mmol) was added and stirring was resumed for additional 12 h. To this mixture copper(ii) triflate (16.27 mg, 0.015 mmol) was added and after 30 min of stirring triethylamine (2.27 mL, 0.015 mmol), the corresponding maleimide **2** (0.05 mmol) and imine **3** (0.05 mmol) were consecutively added. The resulting mixture was stirred for 6 d. Then, the reaction mixture was extracted with dichloromethane (3 × 10 mL) by means of an ultrasound bath. The three organic layers were collected and washed with brine (saturated solution) and water. The resulting organic layer was dried with anhydrous magnesium sulfate. Evaporation of the solvent under reduced pressure furnished the crude cycloadduct 5 as white solid or colourless oil, which was purified by trituration with diethyl ether.

## Conflicts of interest

There are no conflicts to declare.

## Supplementary Material

Supplementary information

Crystal structure data
