# Supplementary material for: Catalysis of a 1,3-dipolar reaction by distorted DNA incorporating a heterobimetallic platinum(ii) and copper(ii) complex
Source: Chem Sci. 2017 Aug 22;8(10):7038–46. doi: 10.1039/c7sc02311a (PMC5637124; doi:10.1039/c7sc02311a)
Supplement: Supplementary file 1 [file SC-008-C7SC02311A-s001.pdf]

Electronic Supplementary Material (ESI) for Chemical Science.
This journal is © The Royal Society of Chemistry 2017

Catalysis of a 1,3-Dipolar Reaction by Distored DNA Incorporating a Heterobimetallic Platinum (II) and Copper (II)

Electronic Supplementary Information

Iván Rivilla[a], Abel de Cózar[a,b], Thomas Schäfer [b,c], Frank J. Hernandez[c], Alexander M. Bittner[b,d], Aitziber Eleta-Lopez[d] Ali Aboudzadeh,[c] José I. Santos[e], José I. Miranda[e] and Fernando P. Cossío[a,*]

[a] Department of Organic Chemistry I, Centro de Innovación en Química Avanzada (ORFEO-CINQA), Universidad del País Vasco/Euskal Herriko Unibertsitatea (UPV/EHU) and Donostia International Physics Center (DIPC). Pº Manuel Lardizabal 3, E-20018 Donostia/San Sebastián, Spain.

[b] Ikerbasque, Basque Foundation for Science Mª Díaz de Haro 3, E-48013 Bilbao, Spain.

[c] NanoBioSeparations Group, POLYMAT University of the Basque Country (UPV/EHU). Avda. Tolosa 72, E-2018 Donostia/San Sebastián, Spain.

[d] CIC NanoGUNE Consolider. Tolosa Hiribidea, 76, E-200018 Donostia/san Sebastián, Spain.

[e] SGIker NMR Facility. Universidad del País Vasco/Euskal Herriko Unibertsitatea (UPV/EHU). Avda. Tolosa 72, E-20018 Donostia/San Sebastián, Spain.

Contents

Commercially available reagents and solvents……………………………….S3

Table S1. Control experiments for the **3a**+**4a**$\rightarrow$**5aa** reaction…………S3

Computational methods and results……………………………………..…..S4

NMR data of **1a** and cycloadducts **5aa-bf** …………………………………..…..S8

Copies of NMR spectra ………………………………………………..………….…...S11

ORTEP diagram of cycloadducts **5bb** and **5bf**……………………………..…...S19

Cartesian coordinates of the QM partition of stationary points……..S20

References for Supplementary Information…………………………..……S46

Commercially available reagents and solvents

Guanosine 5′-monophosphate disodium salt hydrate, $K_2[PtCl_4]$, Lambda phage DNA, methylated from *Escherichia coli* host strain W3110 lyophilized powder, copper(II) trifluoromethanesulfonate, deoxyribonucleic acid sodium salt from salmon testes, *N*-pheylmaleimide and *N*-methylmaleimide, were purchased from Sigma-Aldrich and stored under appropriate conditions. Dichloromethane was purchased from Lab-Scan. 2,2´-Bipyrimidine was purchased from Alfa Aesar. Triethylamine and DMSO were purchased from Panreac. Hexane and Ethyl Acetate for chromatography column were purchased from Scharlab.

Table S1. Control experiments for the **3a**+**4a**→**5aa** reaction (See Scheme 2 of the main text).

Entry	Bipym[a]	$PtCl_2$	$Cu(OTf)_2$	TEA[b]	DNA[c]	Result[d]
1	--	--	--	--	--	n. r.
2	Yes	--	--	--	--	n. r.
3	--	Yes	--	--	--	n. r.
4	--	--	Yes	--	--	n. r.
5	--	--	--	Yes	--	n. r.
6	--	--	--	--	Yes	n. r.
7	Yes (**1a**)[e]	Yes (**1a**)[e]	--	--	--	n. r.
8	Yes (**1a**)[e]	Yes (**1a**)[e]	Yes	--	--	n. r.
9	Yes (**1a**)[e]	Yes (**1a**)[e]	Yes	Yes	--	n. r.
10	Yes (**1a**)[e]	Yes (**1a**)[e]	Yes	Yes	Yes	**5aa**[f]
11	Yes	--	Yes	--	--	n. r.
12	Yes	--	Yes	Yes	--	n. r.
13	Yes	--	Yes	Yes	Yes	n. r.
14	--	Yes	--	--	Yes	n. r.
15	--	Yes	Yes	Yes	Yes	n. r.
16	--	--	Yes	--	Yes	n. r.
17	--	--	Yes	Yes	Yes	n. r.
18	--	--	--	Yes	Yes	n. r.

[a]Bipym: 2,2′-bipyrimidine. [b]TEA: triethylamine. [c]Salmon sperm DNA. [d]n. r.: no (3+2) cycloaddition leading to **5aa** was observed after 6 days of reaction at room temperature. [e]The reaction was carried out with previously formed complex **1a**. [f]Formation of racemic (3+2) *endo*-cycloadduct **5aa** was observed (see Table 1 of the main text).

Computational results

The X-ray structure of double stranded oligodeoxynucleotide complex d(CCTCTG*G*TCTCC)-d(GGAGACCAGAGG) (pdb code: 1aio), was chosen as starting point. The high level DFT layer of the ONIOM calculations included N-methyl maleimide, 2,2'-bipyrimidine and **4a** as well as Cu(II) and Pt(II) cations. Only the guanine units directly connected to the Pt(II) atom were included in the high level layer, whereas all the remaining nucleotides of the DNA helix were considered in the low MM layer. In addition, 22 Na(+1) cations and 509 discrete water molecules were introduced in the low level layer in order to stabilize the whole system (See Figure S1). Preliminary calculations showed that this explicit environment was required to avoid a collapse of the system during the optimization process.

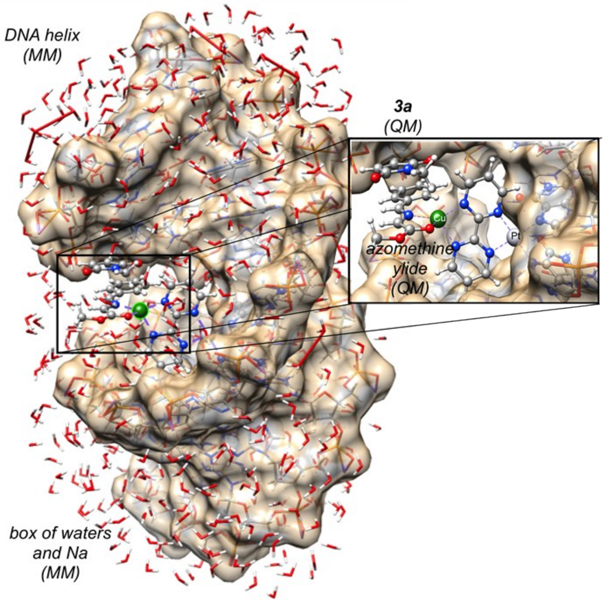

Figure S1. Representation of the different layers used in the ONIOM calculations of *endo*-**RC**. High (QM) and low (MM) level atoms in the ONIOM partitions are represented as ball & stick and tube models respectively. Water molecules and Na(+) cations, treated at the MM level, are represented in wire mode.

In the initial part of the computational study, the structure of azomethine ylide **INT2** was analyzed. Because of the pseudo-tetrahedral environment of the cooper atom, two possible conformers **INT2** and **INT2'** were considered. The calculations showed a clear preference of **INT2** over its **INT2'** counterpart. Single-point calculations of the backbone reflected an energetic difference of ca. 2 kcal mol^{-1}, which suggested that intensive exploration of the possible conformers should result in closer energies for both configurations.

In order to avoid misleading results on the computed activation energies, we ensured that reactive, transition structures and products presented a similar water environment along the possible reaction coordinates. Therefore, only reactive and

product complexes directly connected via the corresponding transition structures by intrinsic reaction coordinate (IRC) calculations were considered.

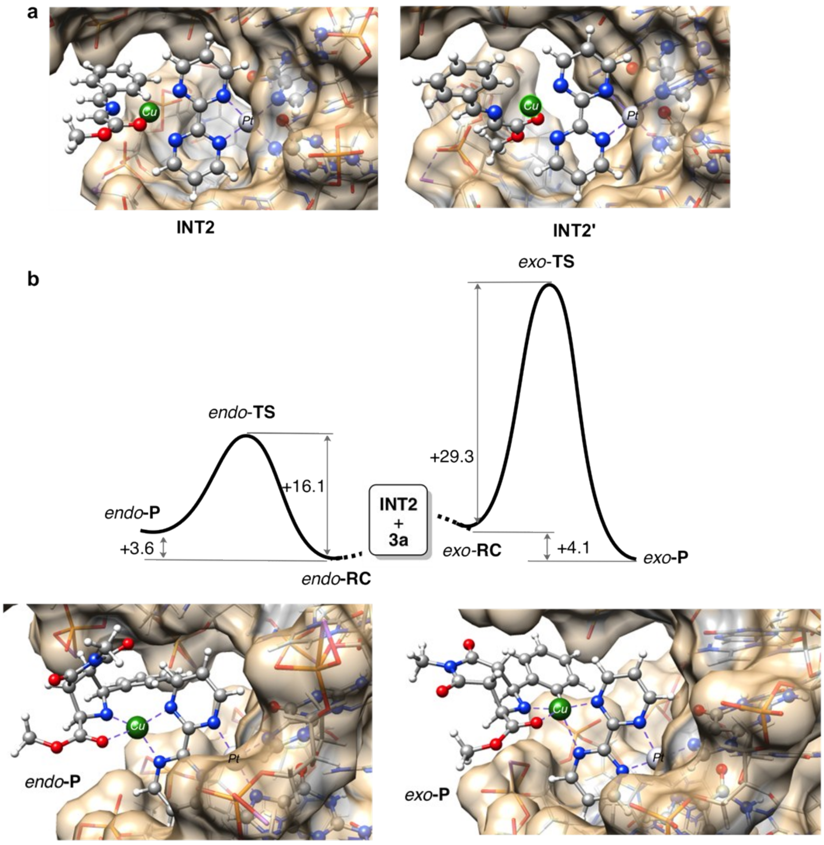

Figure S2. (a) Fully optimized geometries of azomethine ylides **INT2** and **INT2'**. (b) Reaction profiles associated with the (3+2) cycloaddition of **INT2** and **3a**. Calculations were performed at the B3LYP/LanL2DZ:UFF level of theory. Relative energies are in kcal mol^{-1} and were obtained from data reported in Table S2.

In addition, in order to verify the effectiveness of the DNA-catalytic system developed, we computed the activation barriers associated with two different model systems without the DNA scaffold. In the first system we considered the azomethine ylide coordinated to copper and bipyridine as reactive system (Figure S3a). The second model system also includes diaqua platinum in the catalytic system (Figure S3b). In both models, the system was solvated by a box of MM water molecules, and *endo*- and *exo*- approaches were considered.

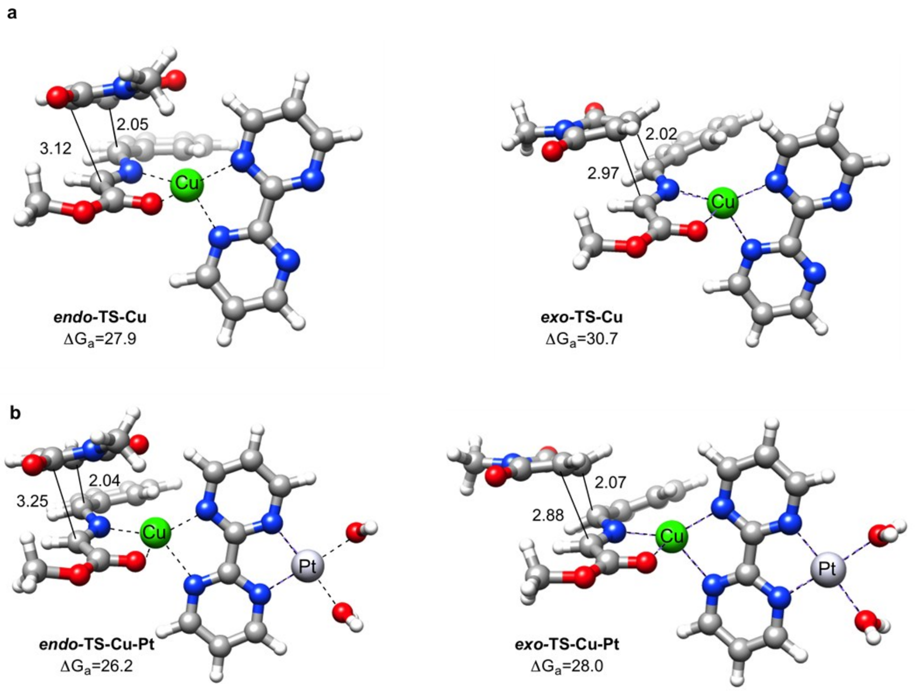

Figure S3. (a) Fully optimized geometries of transition structures associated with the *endo*- and *exo*- (3+2) cycloadditions between azomethine ylide associated with imine 4a and N-methyl maleimide in the presence of the Cu(II)-bipym complex. (b) Same as (a) but with an additional Pt(II)$(H_2O)_2$ unit. Calculations were performed at the B3LYP/LanL2DZ:UFF level of theory. Activation energies are given in kcal mol^{-1} and were obtained from data reported in Table S3.

Our calculations show that in both cases, the computed activation barriers are higher than those computed for the DNA-catalytic system. In addition, the *exo* approaches are disfavored compared to their *endo*- counterparts, as it occurred in the presence of DNA.

Table S2. Total electronic energies[a] (E, in a.u.), zero point vibrational energies[b] (ZPVE, in a. u.), Thermal corrections to Gibbs free energies[c] (TCGFE, in a.u.), and number of imaginary frequencies[d] (NIMAG) of all the stationary points included in figure S2.

Structure	E	ZPVE	TCGFE	NIMAG(ν)
**INT2**	-2515.199041 (-2509.473451)[d]	31.880089	24.277696	0
**INT2'**	-2514.684528 (-2509.469457)[d]	31.844412	24.244623	0
**endo-RC**	-2913.610189	18.347023	14.505919	1 (-4.0716)
**exo-RC**	-2916.603381	18.314116	14.407497	0
**endo-P**	-2913.608963	18.351548	14.516041	0
**exo-P**	-2916.613338	18.317510	14.409977	0
**endo-TS**	-2913.585204	18.347673	14.509305	1 (-347.8835)
**exo-TS**	-2916.578814	18.336136	14.476855	1 (-342.8258)

[a]Computed at the ONIOM(B3LYP/LANL2DZ:UFF) level. [b,c]Computed at 298.15 K at the ONIOM(B3LYP/LANL2DZ:UFF) level. [d]If NIMAG=1, the imaginary frequency ν (in parentheses) is given in cm^{-1}. [e]Single point without considering the water + Na environment.

Table S3. Total electronic energies[a] (E, in a.u.), zero point vibrational energies[b] (ZPVE, in a. u.), thermal corrections to Gibbs free energies[c] (TCGFE, in a.u.), and number of imaginary frequencies[d] (NIMAG) of all the stationary points included in Figure S3.

Structure	E	ZPVE	TCGFE	NIMAG(ν)
**endo-RC-(Cu-bipym)**	-1714.524234	2.730927	1.947269	0
**exo-RC-(Cu-bipym)**	-1714.522641	2.687094	1.917426	0
**endo-P-(Cu-bipym)**	-1714.535620	2.738420	1.965040	0
**exo-P-(Cu-bipym)**	-1714.535751	2.693210	1.931002	0
**endo-TS-(Cu-bipym)**	-1714.486532	2.731979	1.954134	1 (-559.7746)
**exo-TS-(Cu-bipym)**	-1714.473720	2.686365	1.917462	1 (-562.5817)
**endo-RC-(Cu-Pt-bipym)**	-1985.946639	3.215388	2.292364	0
**exo-RC-(Cu-Pt-bipym)**	-1985.854892	3.184986	2.189465	0
**endo-P-(Cu-Pt-bipym)**	-1985.939321	3.220902	2.311317	0
**exo-P-(Cu-Pt-bipym)**	-1985.920086	3.198087	2.297976	0
**endo-TS-(Cu-Pt-bipym)**	-1985.907092	3.215253	2.294630	1 (-590.8853)
**exo-TS-(Cu-Pt-bipym)**	-1985.890064	3.178444	2.269281	1 (-540.7704)

[a]Computed at the ONIOM(B3LYP/LANL2DZ:UFF) level. [b,c]Computed at 298.15 K at the ONIOM(B3LYP/LANL2DZ:UFF) level. [d]If NIMAG=1, the imaginary frequency ν (in parentheses) is given in cm^{-1}.

Table S4. NMR data of **1a** and selected derivatives shown in Figure 2 of the main text.

**Compound**	**Nucleus**	**δ (ppm)**
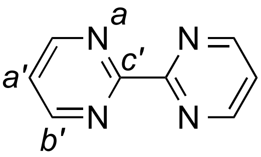	^{15}N	106.18 (a)
	^{13}C	123.63 (a'); 153.67 (b'); 158.86 (c')
**1a** 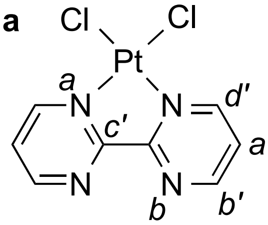	^{15}N	106.26 (b); 109.96 (a)
	^{13}C	124.0 (a´); 149.94, 152.87* (d´); 154.52, 157.51* (c´), 157.29, 161.39* (b´)
**5'-GMP** 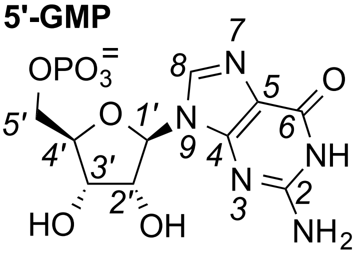	^{15}N	65.61 (NH_2); 145.52 (NH); 149.08 (N3); 159.14 (N7); 164.48 (N9)
	^{13}C	64.37, 63.38* (5´); 71.80, 70.89* (2´); 77.21, 76.35* (3´); 82.91 (4´); 85.84, 84.71* (1´); 113.89 (4); 136.01, 137.67* (8), 149.35, 150.05* (5); 153.28 (2); 154.16, 157.56* (6)
**6** 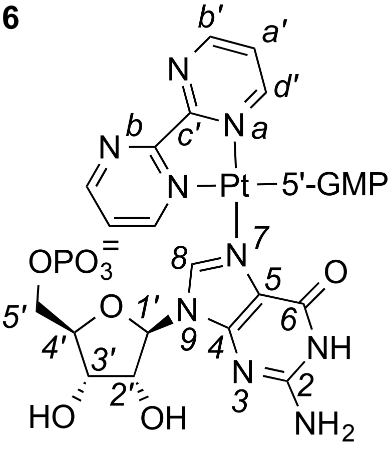	^{15}N	75.74 (NH_2); 106.48 (b); 109.53 (a); 137.05 (NH); 160.02 (N3); 167.24 (N9); 222.73 (N7)
	^{13}C	62.59 (5´); 71.80, 70.81 (2´); 74.61 (3´); 83.21 (4´); 87.98 (1´); 112.80, 113.93* (4); 123.65, (a´), 124.58* (a´), 134.97 (8) (5); 139.25 (5); 151.02 (2), 159.09 (6)

- Signals associated with presence of different conformers in solid state.

In the ^{13}C MAS-NMR spectrum of **5'GMP** all the signals were duplicated due to the existence of two conformers. The assignment of the signals was performed according to previously published work.[1] Davis et al.[2] Proposed that Guanosine interacts with K^+ and Cs^+ giving rise to only the *cis* conformer. It is noticeable that the *anti* conformer is supposed to be the major one in the solid state. Our results shown that the reaction of Guanosine with Pt(bipym)Cl_2 implies a shift of the *C*8 and *C*1' signals to higher fields due to interactions of the former compound with the metallic centre. Moreover, the duplicity of the Guanosine signals on the MAS-NMR spectra disappear when Pt(bipym)Cl_2 **1a** was added. From these data we propose that the rotational freedom about *N*9-*C*1' is broken due to the metal coordination. Therefore, only one of the conformers can be formed.

Characterization data for cycloadducts 5aa-bf.

The ^1H-NMR data for compounds **5aa-bf** were in good agreement with those reported in the literature.[3,4]

Methyl 5-methyl-4,6-dioxo-3-phenyloctahydropyrrolo[3,4-c]pyrrole-1-carboxylate (5aa): White solid, 3.02 mg, yield 21 %. ^1H NMR (400 MHz, Chloroform-*d*) δ 7.38 (bs, 5H) 4.53 (d, *J* = 8.6 Hz, 1H), 4.09 (d, *J* = 6.8 Hz, 1H), 3.91 (s, 3H), 3.60 (d, *J* = 7.2 Hz, 1H), 3.47 (t, *J* = 8.1 Hz, 1H), 2.91 (s, 3H) 2.45 (s, 1H).

Methyl 4,6-dioxo-3,5-diphenyloctahydropyrrolo[3,4-c]pyrrole-1-carboxylate (5ba): White solid, 6.04mg, yield 37%. ^1H NMR (400 MHz, Chloroform-*d*) δ 7.58 – 7.33 (m, 4H), 7.18-7.15 (m, 2H), 7.08 (t, *J* = 8.4 Hz, 2H), 4.63 (d, *J* = 8.8 Hz, 1H), 4.17 (d, *J* = 6.6 Hz, 1H), 3.90 (s, 3H), 3.75 (t, *J* = 7.2 Hz, 1H), 3.59 (t, *J* = 8.3 Hz, 1H), 2.55 (s, 1H)

Methyl 5-(4-methoxyphenyl)-4,6-dioxo-3-phenyloctahydropyrrolo[3,4-c]pyrrole-1-carboxylate (5ca): White solid, 3.84 mg, yield 20 %. ^1H NMR (400 MHz, Chloroform-*d*) δ 7.47 (d, *J* = 7.4 Hz, 2H), 7.37 (m, 2H), 7.09 (d, *J* = 8.5 Hz, 2H), 6.92 (dd, *J* = 8.5 Hz, 2H), 4.64 (d, *J* = 8.8 Hz, 1H), 4.17 (d, *J* = 6.7 Hz, 1H), 3.90 (s, 3H), 3.81 (s, 3H), 3.74 (t, *J* = 7.2 Hz, 1H), 3.58 (t, *J* = 8.3 Hz, 1H) 2.55 (bs, 1H).

Methyl 4,6-dioxo-5-phenyl-3-(p-tolyl)octahydropyrrolo[3,4-c]pyrrole-1-carboxylate, *5bb*: White solid, 4.19 mg, yield 23 %. ^1H NMR (400 MHz, Chloroform-*d*) δ 7.44-7.35 (m, 5H), 7.19 (d, *J*= 7.73 Hz, 2H,), 6.91 (d, *J*= 8.01 Hz, 2H), 4.59 (d, *J* = 8.7 Hz, 1H), 4.15 (d, *J* = 6.7 Hz, 1H), 3.90 (s, 3H), 3.82 (s, 3H), 3.74 (t, *J* = 7.3 Hz, 3H), 3.54 (t, *J* = 8.3 Hz, 1H), 2.50 (s, 1H).

Methyl 3-(4-methoxyphenyl)-4,6-dioxo-5-phenyloctahydropyrrolo[3,4-c]pyrrole-1-carboxylate (5bc): White solid, 6.65 mg, yield 35 %. ^1H NMR (400 MHz, Chloroform-*d*) δ 7.46-7.35 (m, 4H), 7.19 (d, *J* = 7.8 Hz, 2H), 6.91 (d, *J* = 8.2 Hz, 2H), 4.59 (d, *J* = 8.8 Hz,

1H), 4.15 (d, *J* = 6.6 Hz, 1H), 3.90 (s, 3H), 3.82 (s, 3H), 3.74 (t, *J* = 7.2 Hz, 1H), 3.54 (t, *J* = 8.3 Hz, 1H), 2.50 (s, 1H).

Methyl 3-(4-fluorophenyl)-4,6-dioxo-5-phenyloctahydropyrrolo[3,4-c]pyrrole-1-carboxylate (5bd): White solid, 4.78 mg, yield 26 %. ^{1}H NMR (400 MHz, Chloroform-*d*) δ 7.50 – 7.42 (m, 4H), 7.37 (d, *J* = 7.7 Hz, 2H), 7.17 (m, 2H), 7.08 (t, *J* = 8.5 Hz, 2H), 4.62 (d, *J* = 8.7 Hz, 1H), 4.17 (d, *J* = 6.6 Hz, 1H), 3.90 (s, 3H), 3.74 (t, *J* = 7.2 Hz, 1H), 3.57 (t, *J* = 8.4 Hz, 1H), 2.52 (s, 1H).

methyl 3-cyclohexyl-4,6-dioxo-5-phenyloctahydropyrrolo[3,4-c]pyrrole-1-carboxylate (*5be)*: White solid, 5.47mg, yield 32 %. ^{1}H NMR (400 MHz, Chloroform-*d*) δ 7.49 (m, 2H), 7.49 (m, 1H), 7.31 – 7.27 (dt, 1H, *J* = 7.2 Hz, 2H), 4.09 (d, *J* = 6.5 Hz, 1H), 3.92 (s, 3H), 3.73 (d, *J* = 7.2 Hz, 1H), 3.58 (t, *J* = 8.3 Hz, 1H) 3.07 (m, 1H), 2.48 (m, 1H) 2.04 (m, 1H), 1.82 (m,4H), 1,24 (m, 4H).

Methyl 4,6-dioxo-5-phenyl-3-(thiophen-2-yl)octahydropyrrolo[3,4-c]pyrrole-1-carboxylate (5bf): White solid, 4.81 mg, yield 27 %. ^{1}H NMR (400 MHz, Chloroform-*d*) δ 7.44-7.32 (m, 1H), 7.26-7.15 (m, 3H), 7.02 (d, *J*= 7.25 Hz), 4.91 (d, *J* = 8.6 Hz, 1H), 4.12 (d, *J* = 6.2 Hz, 1H), 3.86 (s, 3H), 3.73 (t, *J* = 7.2 Hz, 1H), 3.55 (t, *J* = 8.7 Hz, 1H), 2.72 (s, 1H)

Copies of NMR spectra

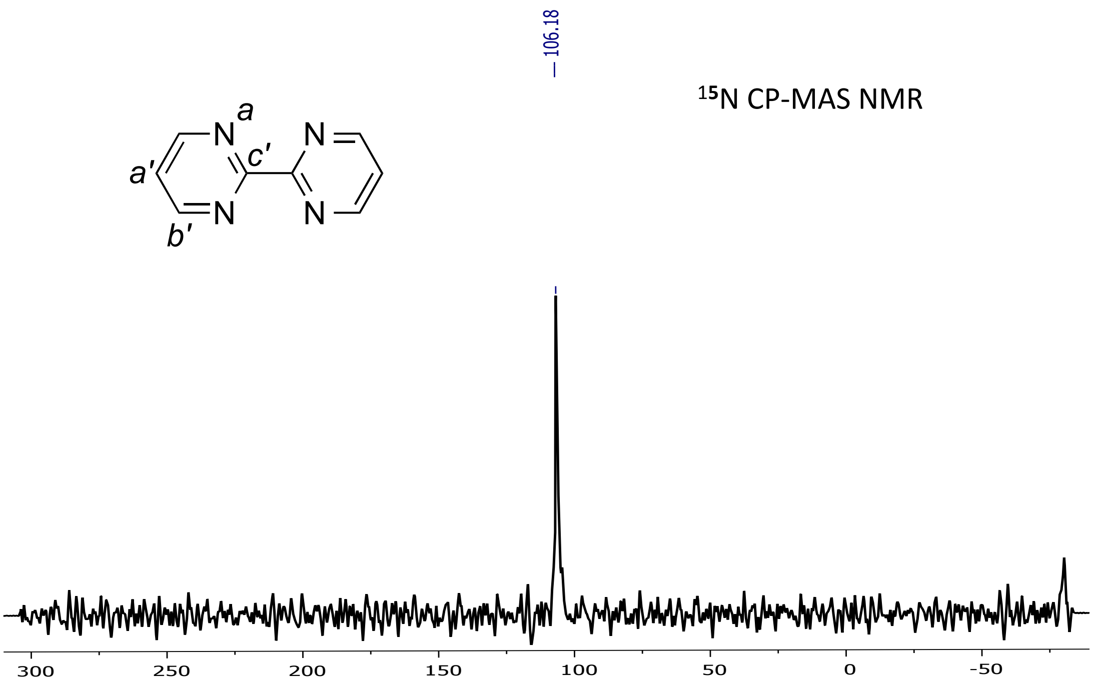

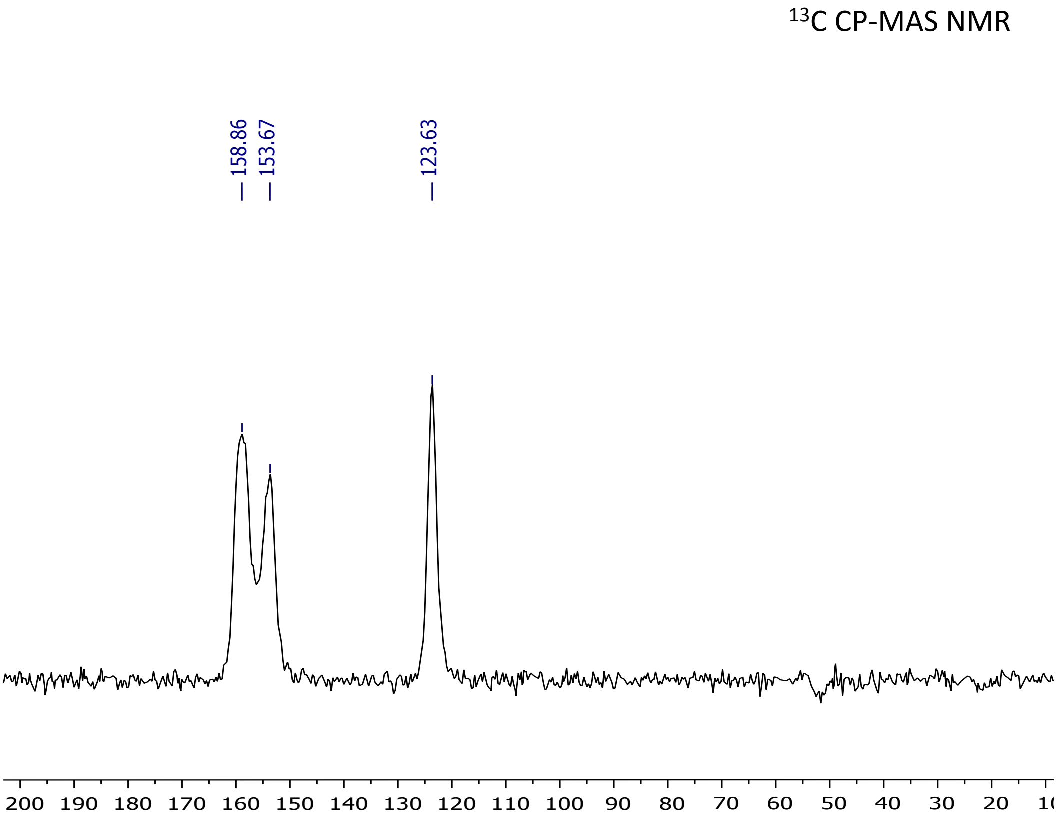

^{15}N CP-MAS NMR

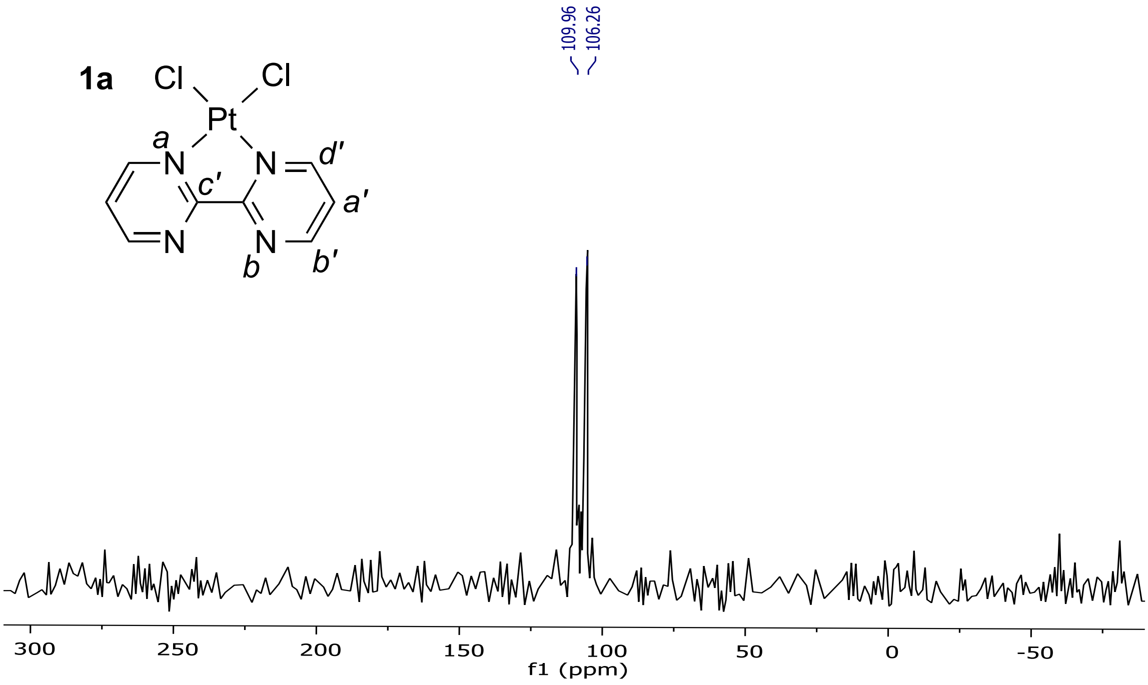

^{13}C CP-MAS NMR

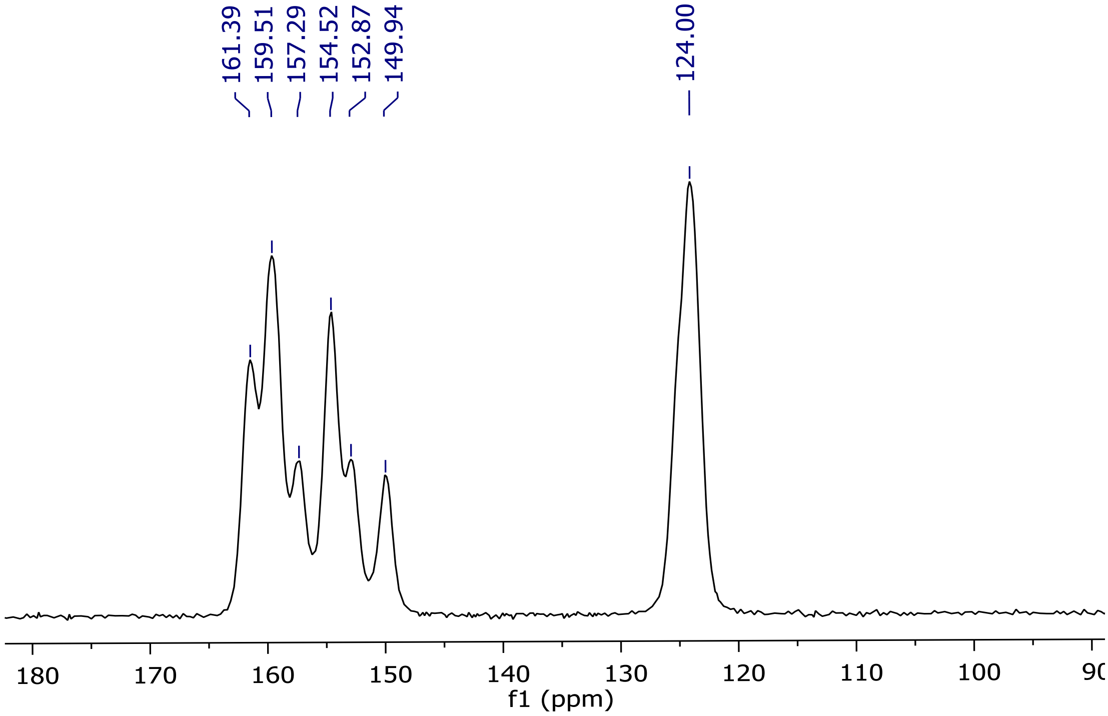

5'-GMP

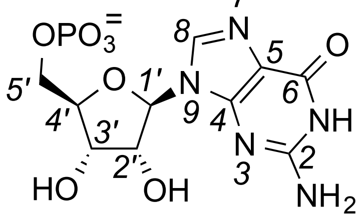
OPO3=
5'
4'
3'
2'
1'
O
HO
OH
N
9
8
7
N
5
6
O
NH
4
N
3
2
NH2

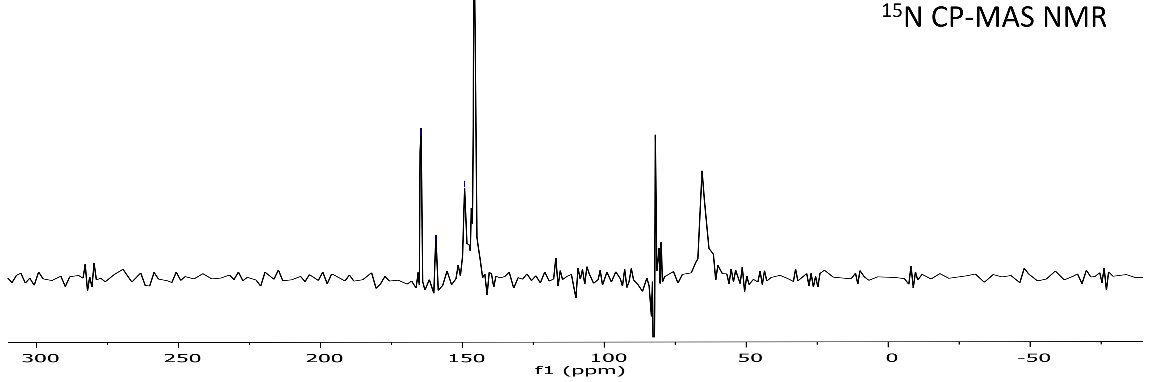
164.48
159.14
149.08
145.52
65.61
15N CP-MAS NMR
300
250
200
150
100
50
0
-50
f1 (ppm)

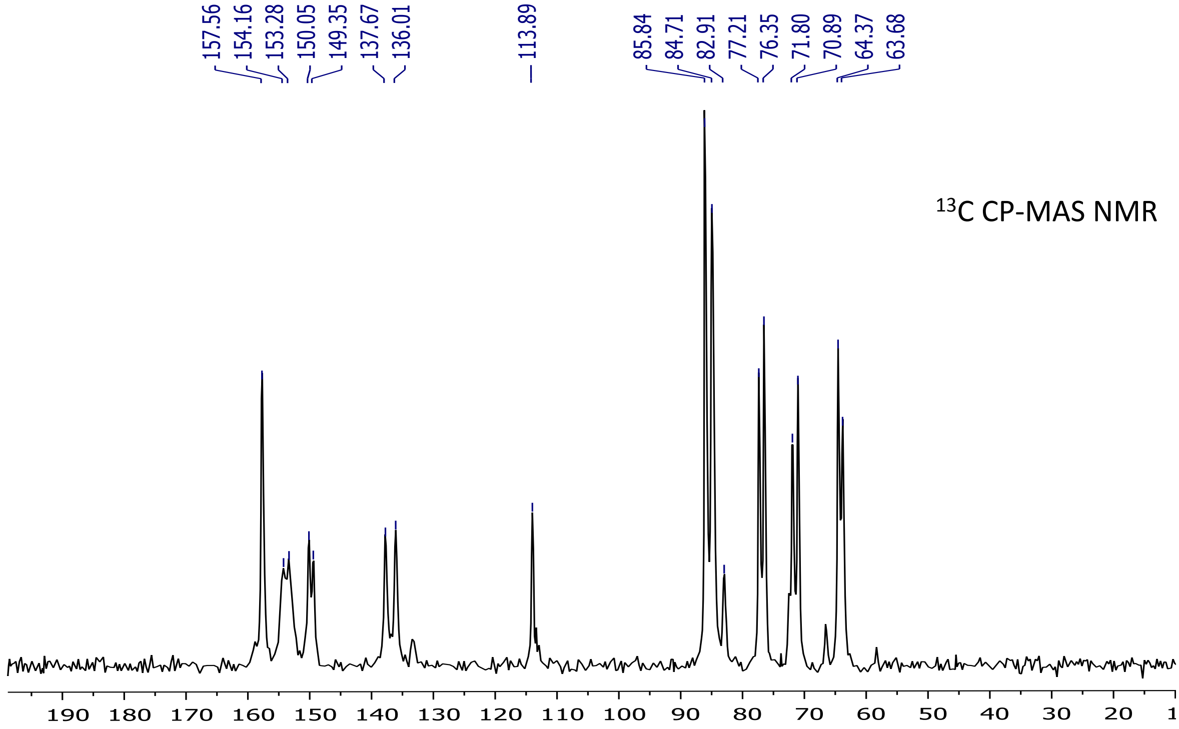
157.56
154.16
153.28
150.05
149.35
137.67
136.01
113.89
85.84
84.71
82.91
77.21
76.35
71.80
70.89
64.37
63.68
13C CP-MAS NMR
190 180 170 160 150 140 130 120 110 100 90 80 70 60 50 40 30 20 1

6

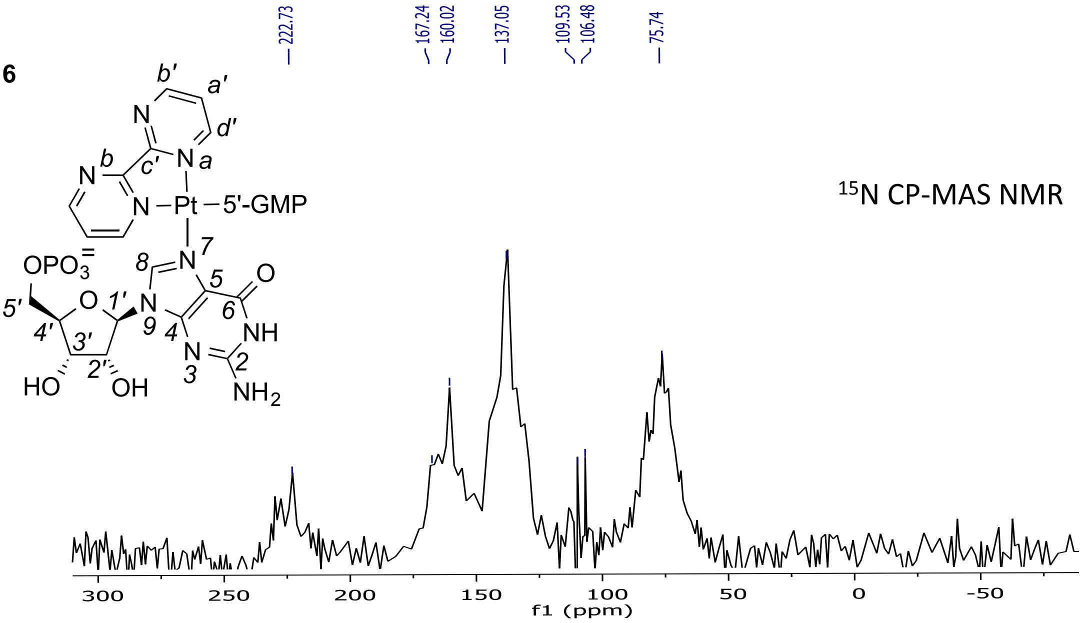

b'
a'
d'
N
c'
N a
b
N
N
Pt
5'-GMP
8
N
7
5
O
OPO$_3^=$
5'
4'
O
1'
N
9
4
6
NH
3'
2'
N
3
2
HO
OH
NH$_2$
^{15}N CP-MAS NMR
222.73
167.24
160.02
137.05
109.53
106.48
75.74
300
250
200
150
100
50
0
-50
f1 (ppm)

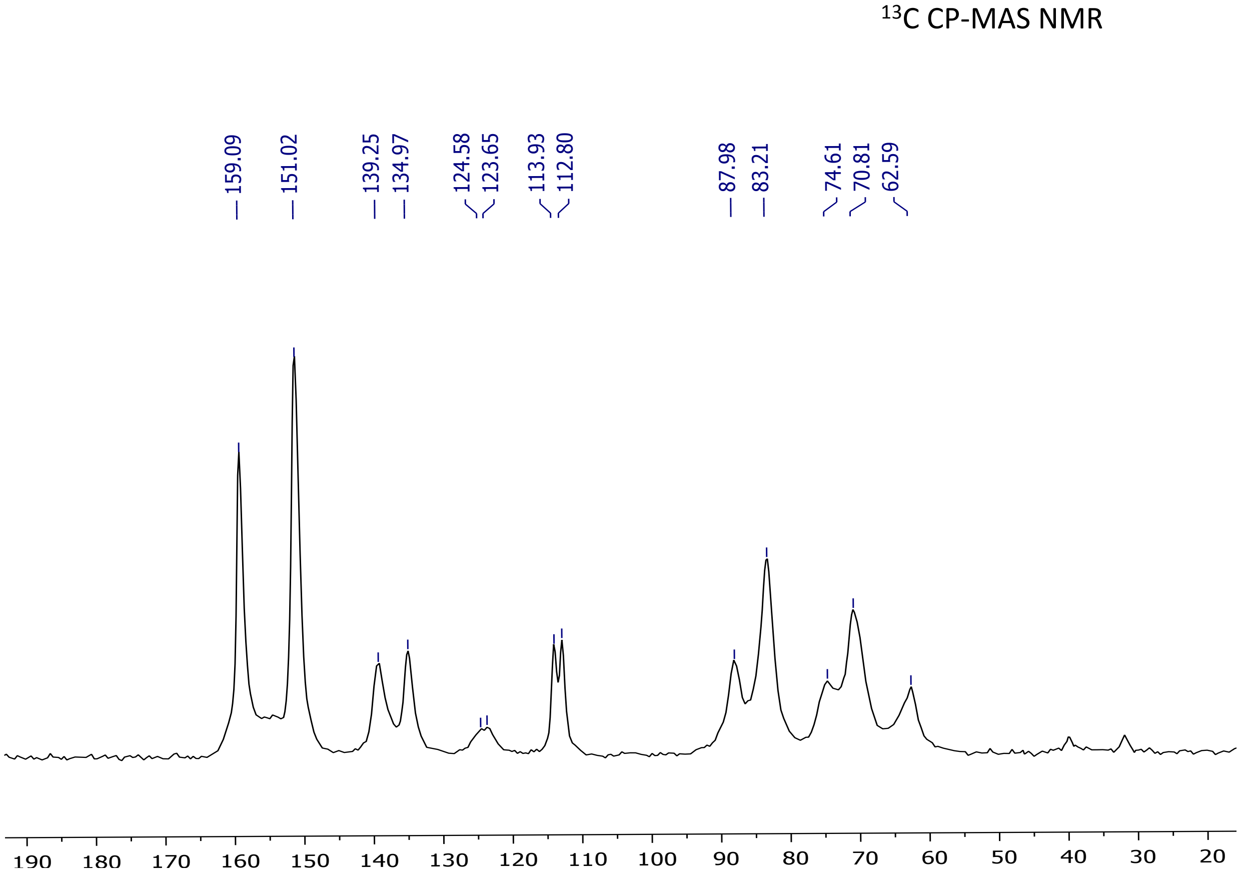

^{13}C CP-MAS NMR
159.09
151.02
139.25
134.97
124.58
123.65
113.93
112.80
87.98
83.21
74.61
70.81
62.59
190
180
170
160
150
140
130
120
110
100
90
80
70
60
50
40
30
20

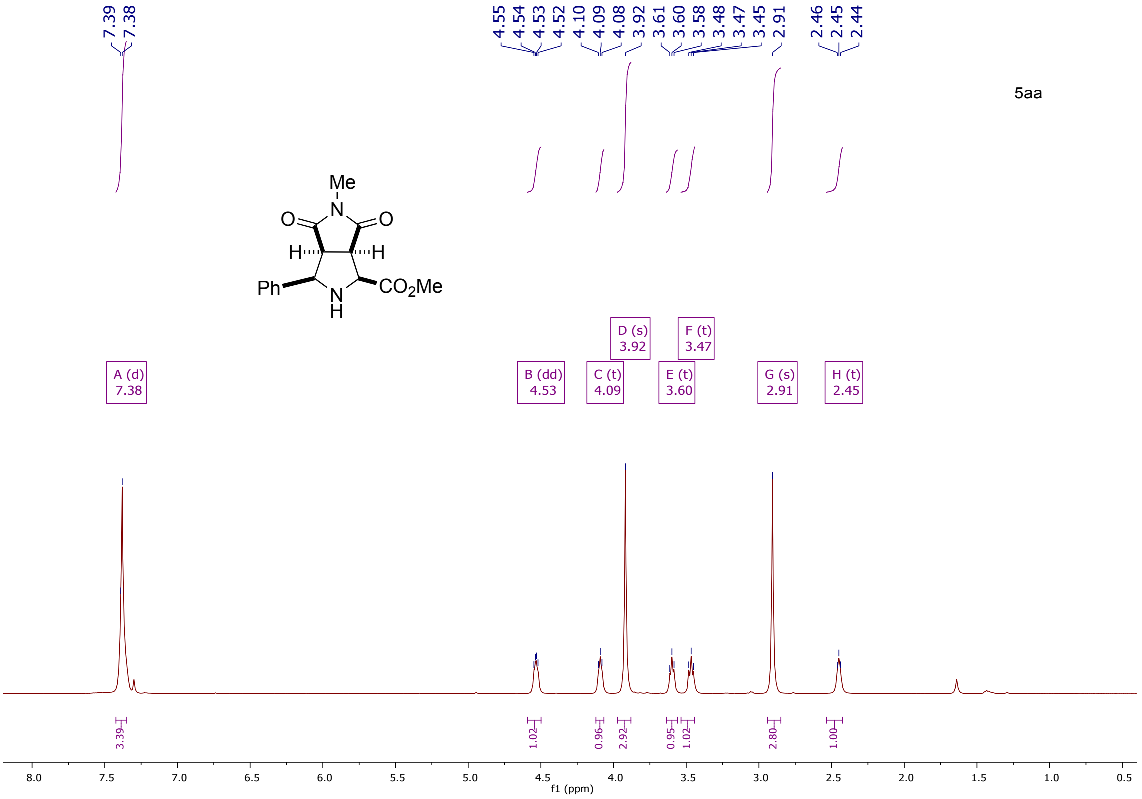

7.39
7.38
4.55
4.54
4.53
4.52
4.10
4.09
4.08
3.92
3.61
3.60
3.58
3.48
3.47
3.45
2.91
2.46
2.45
2.44
Me
N
O
O
H
H
Ph
N
H
CO_2Me
D (s) 3.92
F (t) 3.47
A (d) 7.38
B (dd) 4.53
C (t) 4.09
E (t) 3.60
G (s) 2.91
H (t) 2.45
3.39
1.02
0.96
2.92
0.95
1.02
2.80
1.00
f1 (ppm)

5aa

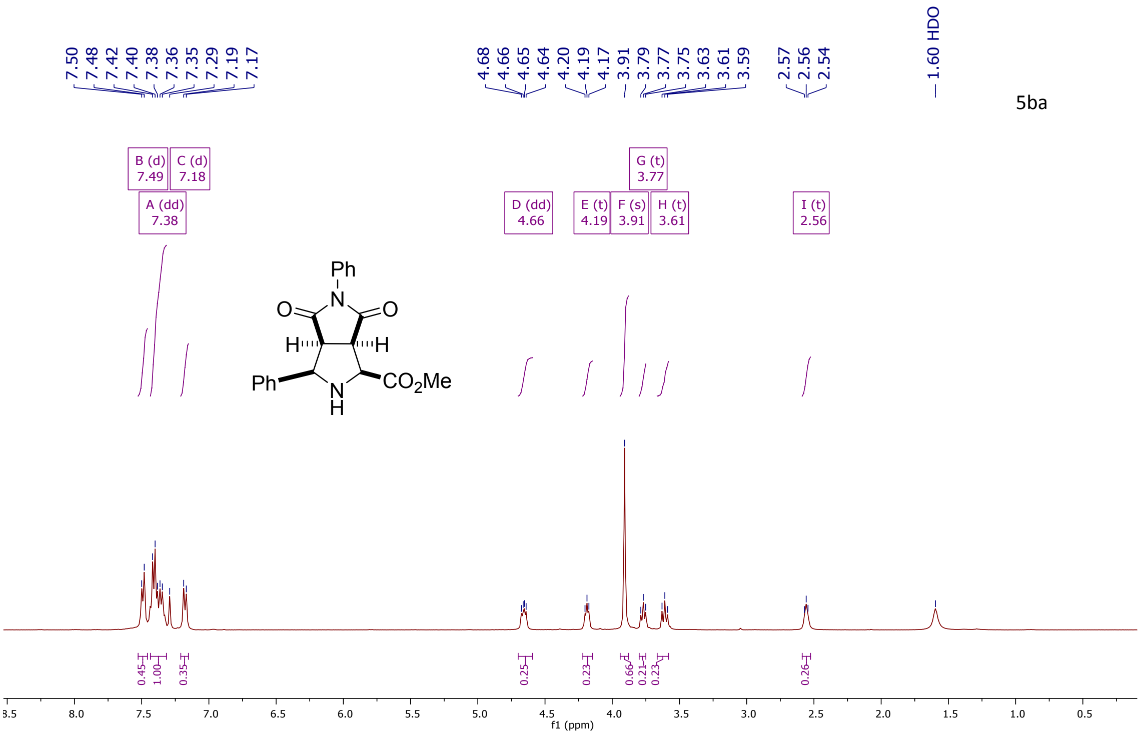

7.50
7.48
7.42
7.40
7.38
7.36
7.35
7.29
7.19
7.17
4.68
4.66
4.65
4.64
4.20
4.19
4.17
3.91
3.79
3.77
3.75
3.63
3.61
3.59
2.57
2.56
2.54
1.60 HDO
B (d) 7.49
C (d) 7.18
A (dd) 7.38
G (t) 3.77
D (dd) 4.66
E (t) 4.19
F (s) 3.91
H (t) 3.61
I (t) 2.56
Ph
N
O
O
H
H
Ph
N
H
CO_2Me
0.45
1.00
0.35
0.25
0.23
0.66
0.21
0.23
0.26
f1 (ppm)

5ba

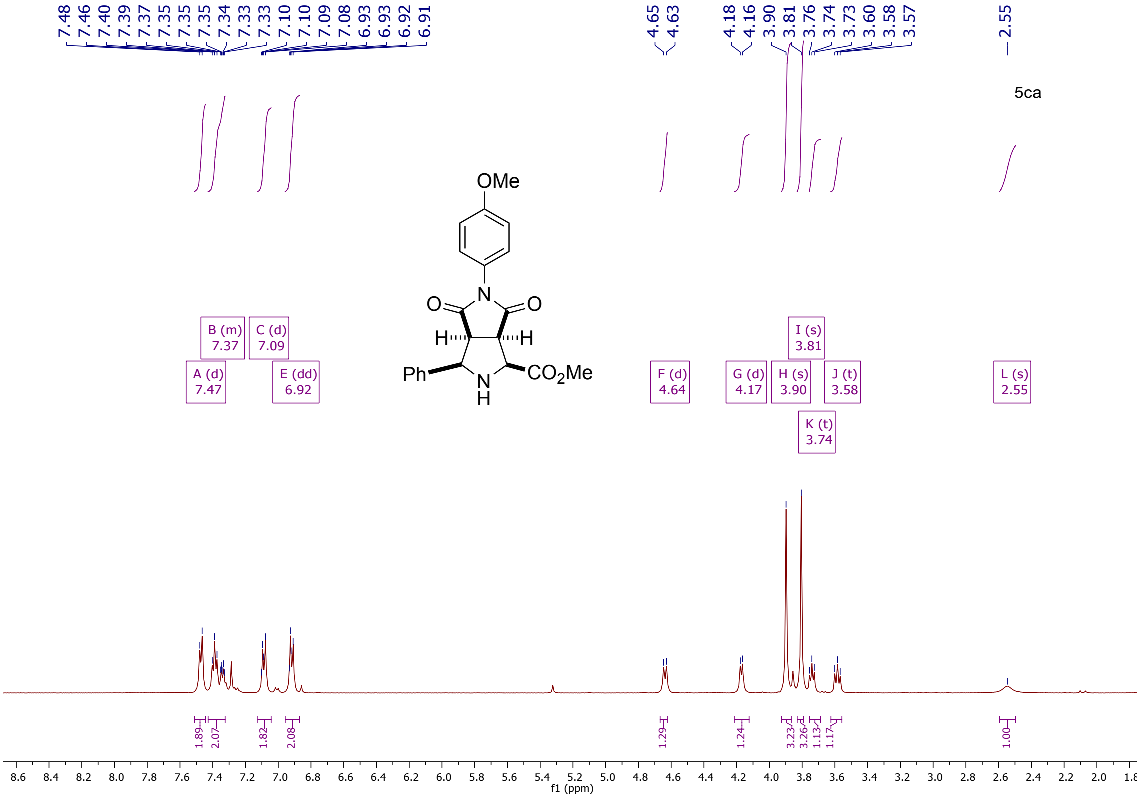
5ca
f1 (ppm)

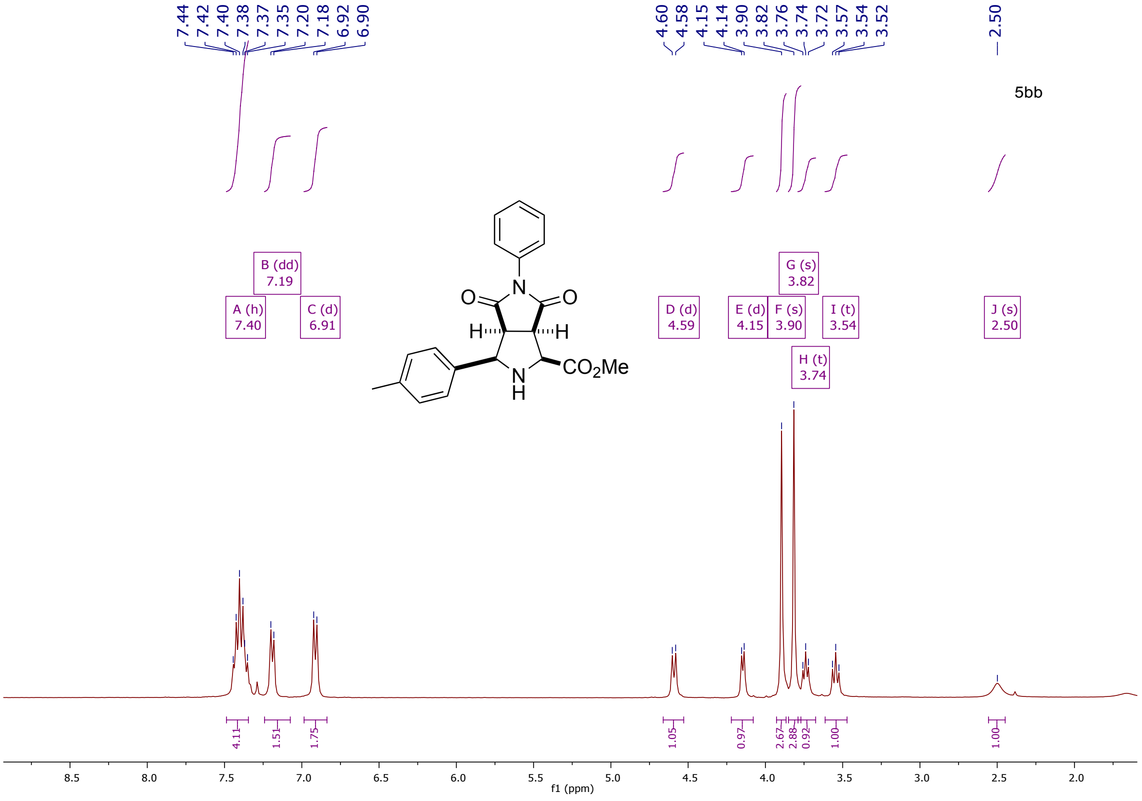
5bb
f1 (ppm)

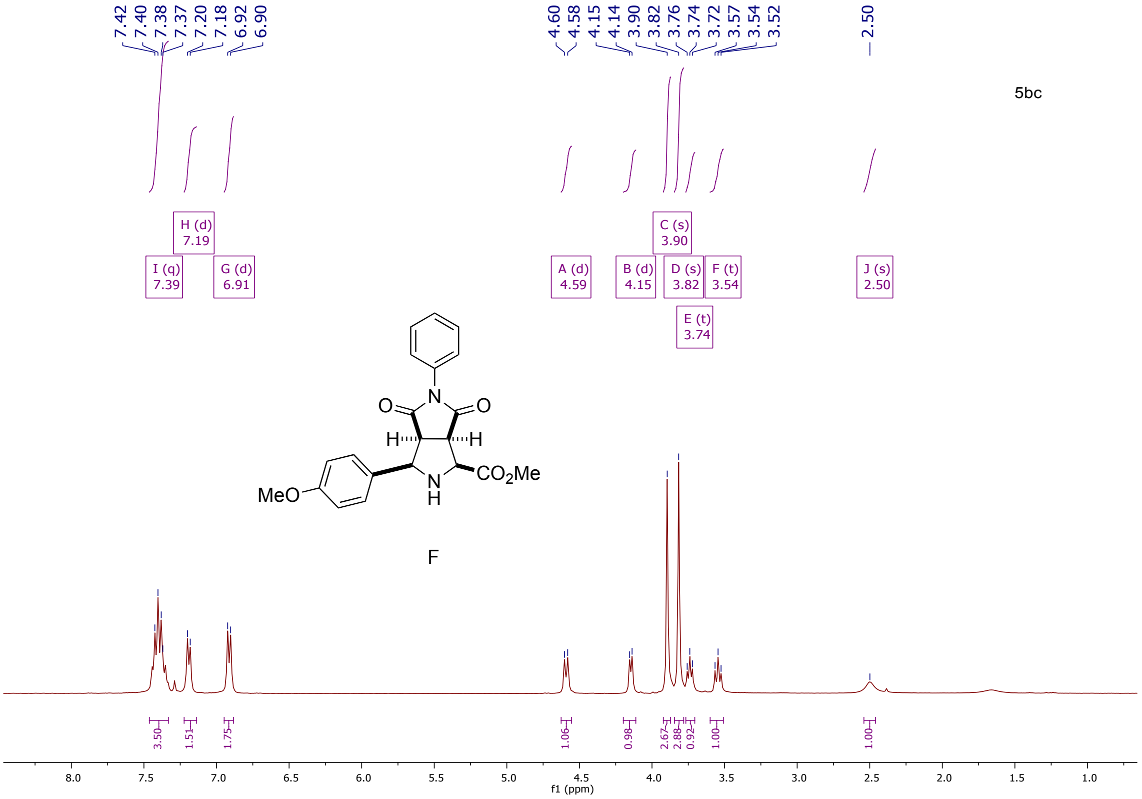

7.42 7.40 7.38 7.37 7.20 7.18 6.92 6.90
4.60 4.58 4.15 4.14 3.90 3.82 3.76 3.74 3.72 3.57 3.54 3.52
2.50
H (d) 7.19
I (q) 7.39
G (d) 6.91
C (s) 3.90
A (d) 4.59
B (d) 4.15
D (s) 3.82
F (t) 3.54
E (t) 3.74
J (s) 2.50
MeO
CO2Me
N
H
O
F
3.50 1.51 1.75 1.06 0.98 2.67 2.88 0.92 1.00 1.00
f1 (ppm)

5bc

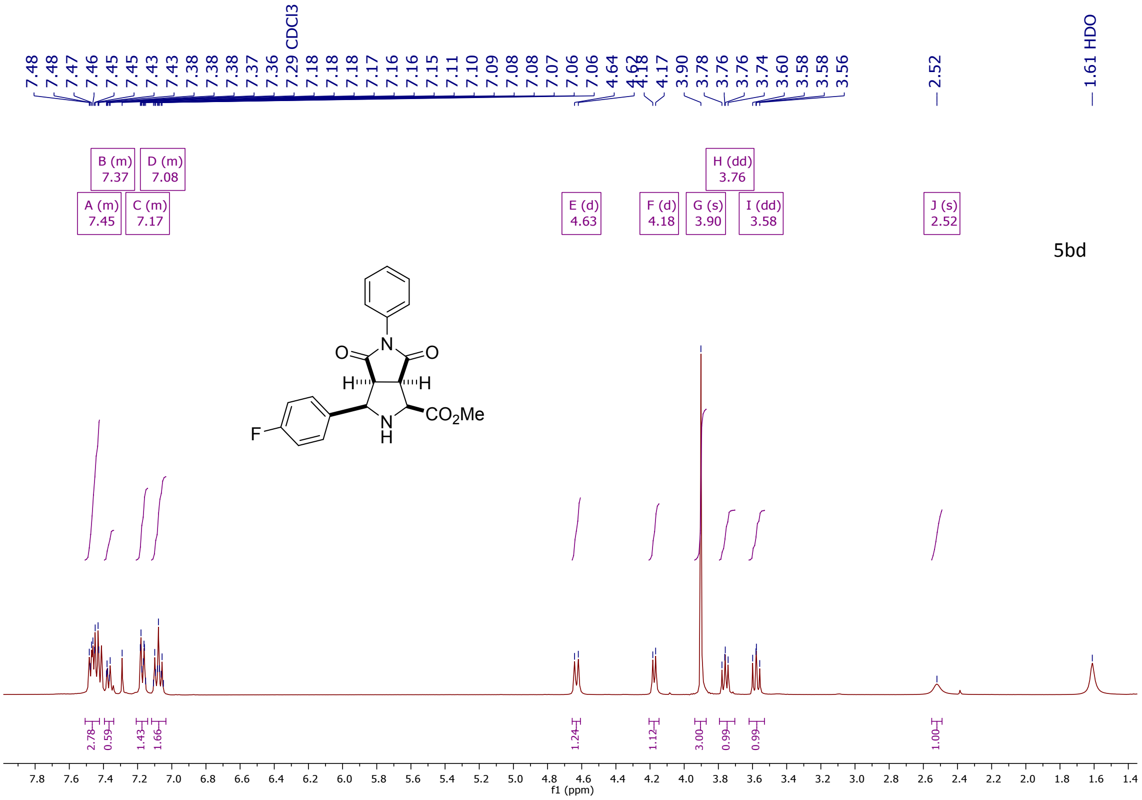

7.48 7.48 7.47 7.46 7.45 7.45 7.43 7.43 7.38 7.38 7.38 7.37 7.36 7.29 CDCl3 7.18 7.18 7.18 7.17 7.16 7.16 7.15 7.11 7.10 7.09 7.08 7.08 7.07 7.06 7.06 4.64 4.62 4.18 4.17 3.90 3.78 3.76 3.76 3.74 3.60 3.58 3.58 3.56
2.52
1.61 HDO
B (m) 7.37
D (m) 7.08
A (m) 7.45
C (m) 7.17
H (dd) 3.76
E (d) 4.63
F (d) 4.18
G (s) 3.90
I (dd) 3.58
J (s) 2.52
F
CO2Me
N
H
O
2.78 0.59 1.43 1.66 1.24 1.12 3.00 0.99 0.99 1.00
f1 (ppm)

5bd

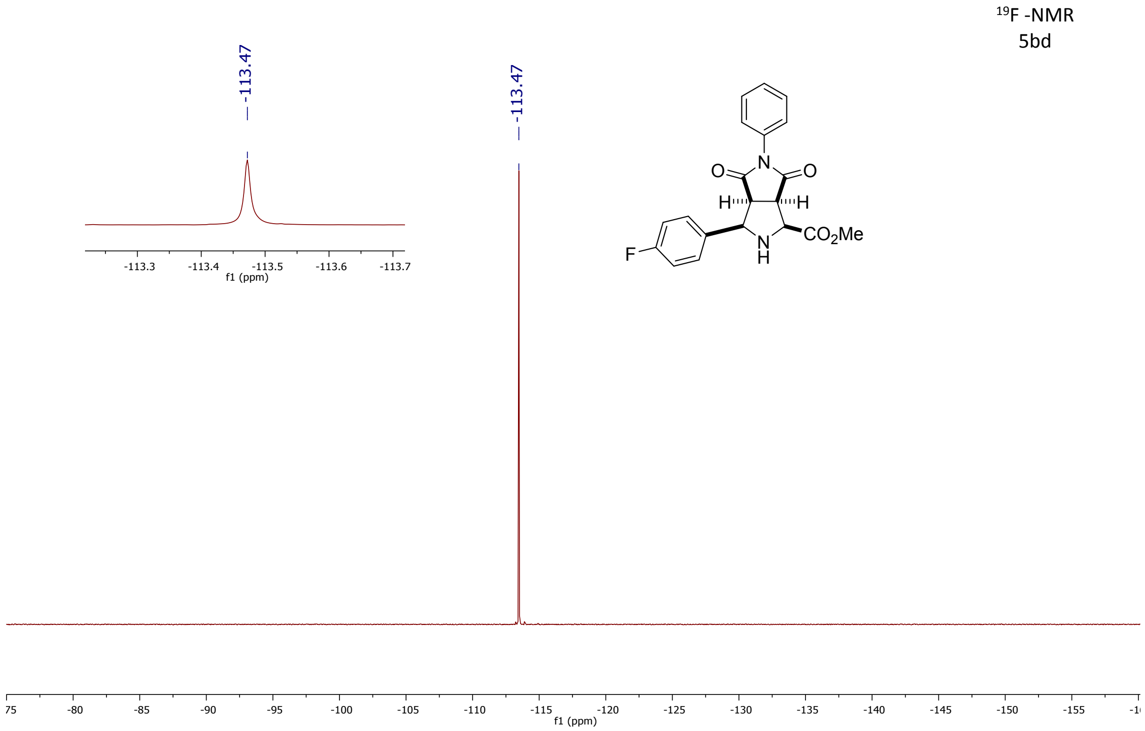

19F -NMR
5bd
-113.47
-113.47
-113.3 -113.4 -113.5 -113.6 -113.7
f1 (ppm)
-75 -80 -85 -90 -95 -100 -105 -110 -115 -120 -125 -130 -135 -140 -145 -150 -155
f1 (ppm)

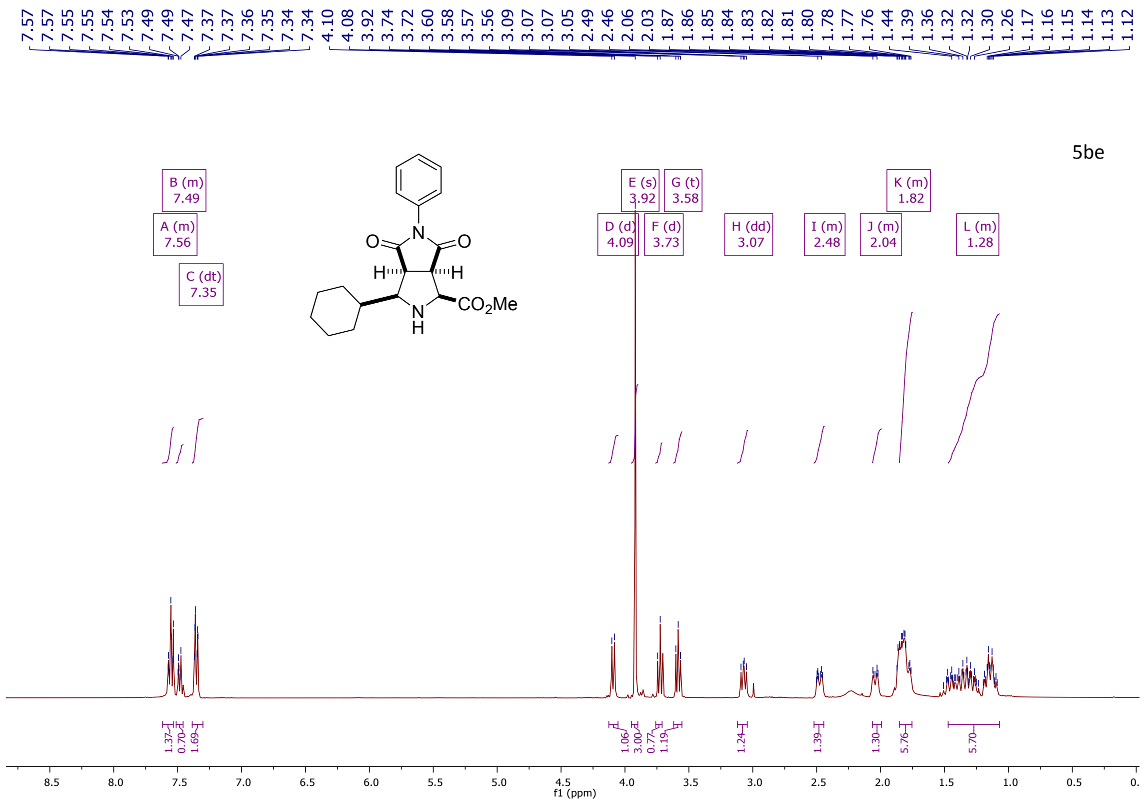

5be
7.57 7.57 7.55 7.55 7.54 7.53 7.49 7.49 7.47 7.37 7.37 7.36 7.35 7.34 7.34 4.10 4.08 3.92 3.74 3.72 3.60 3.58 3.57 3.56 3.09 3.07 3.07 3.05 2.49 2.46 2.06 2.03 1.87 1.86 1.85 1.84 1.83 1.82 1.81 1.80 1.78 1.77 1.76 1.44 1.39 1.36 1.32 1.32 1.30 1.26 1.17 1.16 1.15 1.14 1.13 1.12
A (m) 7.56
B (m) 7.49
C (dt) 7.35
D (d) 4.09
E (s) 3.92
F (d) 3.73
G (t) 3.58
H (dd) 3.07
I (m) 2.48
J (m) 2.04
K (m) 1.82
L (m) 1.28
1.37 0.70 1.69 1.06 3.00 0.77 1.19 1.24 1.39 1.30 5.76 5.70
8.5 8.0 7.5 7.0 6.5 6.0 5.5 5.0 4.5 4.0 3.5 3.0 2.5 2.0 1.5 1.0 0.5 0.
f1 (ppm)

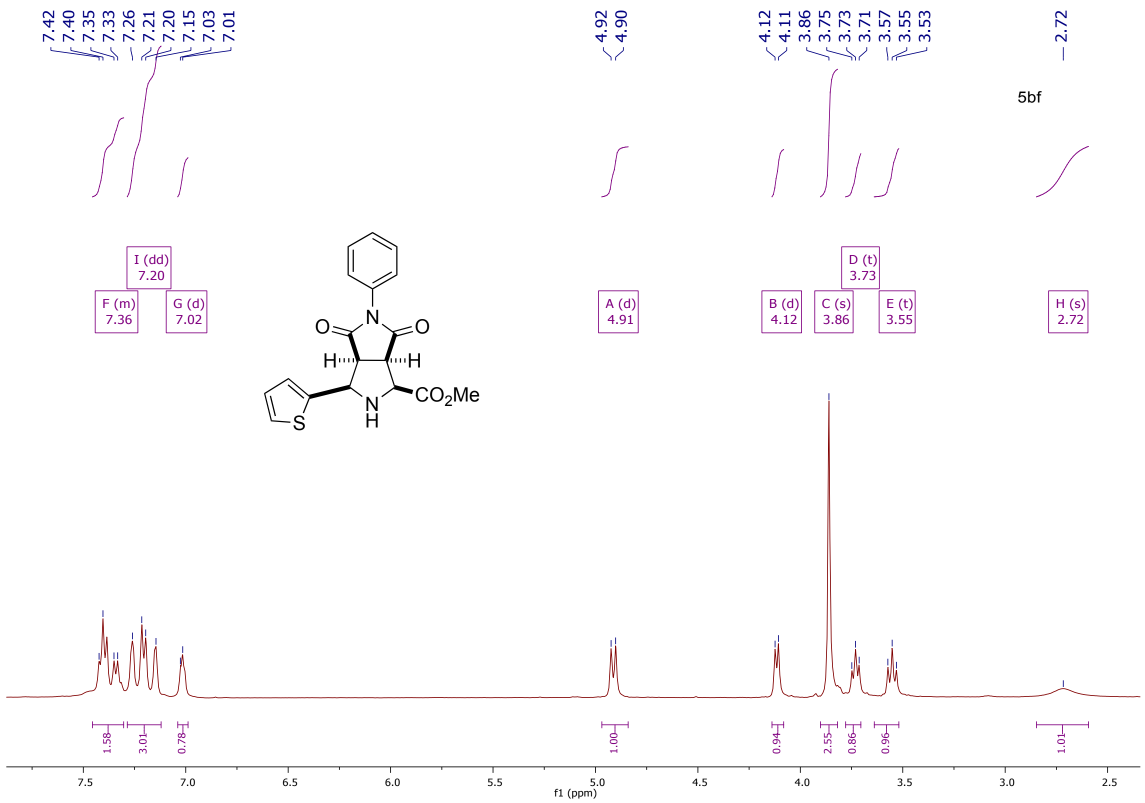

ORTEP diagram of cycloadducts 5bb and 5bf

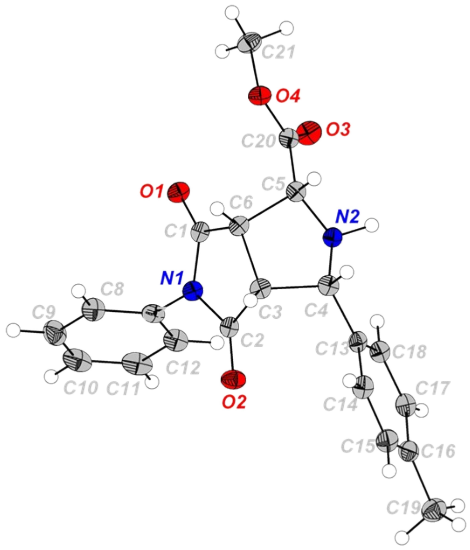

Figure S3. ORTEP drawing at 50% ellipsoid probability of the compound **5bb CCDC 1411373**

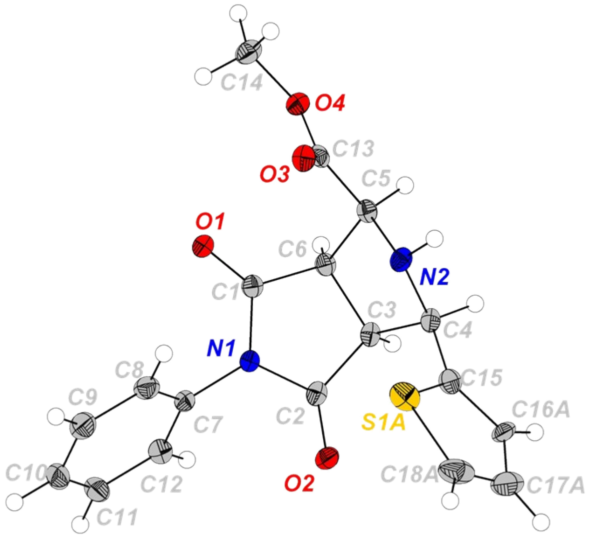

Figure S4. ORTEP drawing at 50% ellipsoid probability of the compound **5bf CCDC 1411372**

Cartesian coordinates of the QM partition of all the stationary points discussed in the main text.

INT2

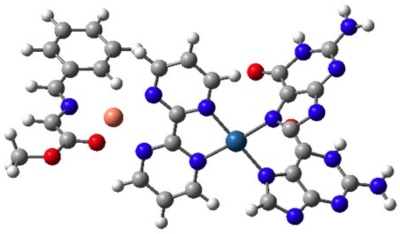

Center Number	Atomic Number	Atomic Type	Coordinates (Angstroms) X	Y	Z
1	7	0	-4.463108	-3.595685	0.453014
2	6	0	-3.199423	-3.211987	0.026182
3	7	0	-2.965528	-1.938760	0.387863
4	6	0	-4.129678	-1.482982	1.046188
5	6	0	-4.464390	-0.218821	1.663195
6	7	0	-5.806785	-0.251401	2.186937
7	6	0	-6.712267	-1.293262	2.054697
8	7	0	-7.983919	-1.123748	2.504329
9	7	0	-6.375489	-2.457863	1.490717
10	6	0	-5.096882	-2.507613	1.041563
11	1	0	-2.514698	-3.846723	-0.512879
12	1	0	-6.063280	0.597368	2.683541
13	1	0	-8.314956	-0.273355	2.935491
14	1	0	-8.611915	-1.913352	2.431753
15	7	0	-4.786106	0.444917	-2.603052
16	6	0	-3.736306	-0.388511	-2.262488
17	7	0	-2.921746	0.220775	-1.389703
18	6	0	-3.433911	1.510366	-1.185932
19	6	0	-2.943053	2.616882	-0.392680
20	8	0	-1.887350	2.723778	0.255349
21	7	0	-3.862995	3.722558	-0.468758
22	6	0	-5.008985	3.777912	-1.246698
23	7	0	-5.731232	4.932393	-1.258003
24	7	0	-5.423373	2.747860	-1.994858
25	6	0	-4.612342	1.658118	-1.933169
26	1	0	-3.591303	4.515193	0.107148
27	1	0	-5.440573	5.770146	-0.775937
28	1	0	-6.562685	4.959252	-1.831919
29	6	0	1.371099	-1.004729	0.346412
30	6	0	0.166992	-2.738025	1.406498
31	6	0	1.363710	-3.223683	1.933152
32	1	0	-0.783773	-3.215397	1.607236
33	6	0	2.571892	-2.522278	1.630769
34	1	0	1.382113	-4.108991	2.553962
35	1	0	3.530189	-2.860001	2.008776
36	6	0	1.338404	0.080244	-0.541929
37	6	0	0.021127	1.449381	-1.938134
38	6	0	1.169064	2.140802	-2.327669
39	1	0	-0.954302	1.693658	-2.329838
40	6	0	2.418730	1.736936	-1.774276
41	1	0	1.107563	2.959660	-3.032608
42	1	0	3.343469	2.237126	-2.041276
43	7	0	0.085216	0.422442	-1.052746
44	7	0	2.506347	0.717049	-0.906227
45	7	0	0.144505	-1.634711	0.607963
46	7	0	2.572980	-1.426768	0.865561
47	7	0	6.144681	-0.071258	0.059449
48	6	0	6.803892	-1.090957	-0.617626
49	6	0	6.030995	-2.008726	-1.355388
50	8	0	4.739625	-1.957607	-1.436328
51	1	0	7.887468	-1.128458	-0.587496
52	6	0	6.834152	0.860835	0.696735

53	6	0	6.312533	2.016490	1.419566
54	6	0	4.934609	2.296347	1.624756
55	6	0	7.260405	2.939956	1.940830
56	6	0	4.531027	3.451299	2.312010
57	1	0	4.185772	1.604744	1.247057
58	6	0	6.854921	4.094101	2.623963
59	1	0	8.322064	2.746790	1.795876
60	6	0	5.484492	4.362041	2.814323
61	1	0	3.470098	3.645399	2.457791
62	1	0	7.609405	4.774790	3.007951
63	1	0	5.165695	5.255176	3.345180
64	1	0	7.927621	0.793580	0.668701
65	8	0	6.629926	-3.054220	-2.059203
66	6	0	8.075942	-3.243631	-1.986776
67	1	0	8.278993	-4.133290	-2.585162
68	1	0	8.612312	-2.384955	-2.410999
69	1	0	8.402827	-3.409634	-0.951918
70	8	0	-3.790378	0.824588	1.780191
71	29	0	4.133086	-0.364129	-0.210464
72	78	0	-1.422523	-0.734678	-0.337089

INT2’

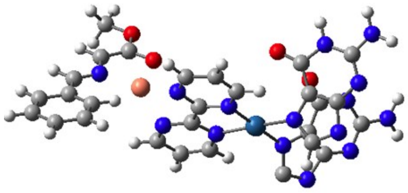

Center Number	Atomic Number	Atomic Type	Coordinates (Angstroms) X	Y	Z
1	7	0	-4.337901	-2.971813	-2.238062
2	6	0	-3.118776	-2.312777	-2.306522
3	7	0	-2.929767	-1.581348	-1.195734
4	6	0	-4.090108	-1.743374	-0.402948
5	6	0	-4.471376	-1.209314	0.886825
6	7	0	-5.792758	-1.654233	1.251641
7	6	0	-6.632119	-2.443271	0.480101
8	7	0	-7.876961	-2.729526	0.944976
9	7	0	-6.256921	-2.928255	-0.708036
10	6	0	-5.001399	-2.575911	-1.080692
11	1	0	-6.085413	-1.330407	2.170856
12	1	0	-8.253268	-2.337088	1.793473
13	1	0	-8.451592	-3.352289	0.393498
14	7	0	-4.819085	2.172375	-1.593403
15	6	0	-3.775275	1.322340	-1.902836
16	7	0	-2.937565	1.205189	-0.863639
17	6	0	-3.435340	2.037057	0.152665
18	6	0	-2.957786	2.309512	1.492067
19	8	0	-1.918618	1.930717	2.060114
20	7	0	-3.869105	3.190744	2.175592
21	6	0	-5.030597	3.732687	1.649714
22	7	0	-5.780520	4.559710	2.431689
23	7	0	-5.441498	3.474545	0.402548
24	6	0	-4.625823	2.635927	-0.290048
25	1	0	-3.658806	0.820424	-2.847215
26	1	0	-3.587569	3.388789	3.131645
27	1	0	-5.469761	4.909738	3.324440
28	1	0	-6.630589	4.934248	2.034171
29	6	0	1.416943	-0.826829	-0.759776
30	6	0	0.224625	-2.795387	-1.292727
31	6	0	1.434015	-3.478052	-1.398269
32	1	0	-0.723321	-3.299004	-1.439221
33	6	0	2.641843	-2.760462	-1.130106
34	1	0	1.463738	-4.523103	-1.669707

35	1	0	3.610183	-3.246228	-1.176252
36	6	0	1.355988	0.540551	-0.443084
37	6	0	-0.014149	2.443356	-0.186168
38	6	0	1.104796	3.192254	0.173920
39	1	0	-0.998183	2.879225	-0.239208
40	6	0	2.360083	2.531275	0.244200
41	1	0	1.011811	4.240058	0.419963
42	1	0	3.254048	3.054884	0.561291
43	7	0	0.088217	1.124941	-0.487601
44	7	0	2.489212	1.230799	-0.061600
45	7	0	0.185517	-1.467689	-0.983038
46	7	0	2.628795	-1.466403	-0.815427
47	7	0	5.799880	-0.580766	1.069495
48	6	0	5.620427	-1.433334	2.154547
49	6	0	4.308189	-1.642347	2.635569
50	8	0	3.255113	-1.099954	2.121081
51	1	0	6.493792	-1.883657	2.616278
52	6	0	7.011386	-0.303889	0.613651
53	6	0	7.368270	0.630794	-0.452326
54	6	0	6.454344	1.489598	-1.120331
55	6	0	8.738023	0.708234	-0.828378
56	6	0	6.893778	2.371394	-2.118635
57	1	0	5.400072	1.459670	-0.851660
58	6	0	9.175900	1.591026	-1.826345
59	1	0	9.458923	0.067379	-0.325292
60	6	0	8.255348	2.432057	-2.481370
61	1	0	6.177958	3.020376	-2.616801
62	1	0	10.229512	1.624386	-2.091945
63	1	0	8.589394	3.118680	-3.254781
64	1	0	7.860882	-0.799415	1.095453
65	8	0	4.066368	-2.468205	3.737906
66	6	0	5.162871	-3.212512	4.348261
67	1	0	4.721446	-3.712049	5.211934
68	1	0	5.566181	-3.955521	3.648350
69	1	0	5.969621	-2.549527	4.686577
70	8	0	-3.852661	-0.450411	1.657997
71	29	0	3.973853	-0.035295	0.408368
72	78	0	-1.408765	-0.195317	-0.861626

----

endo-RC

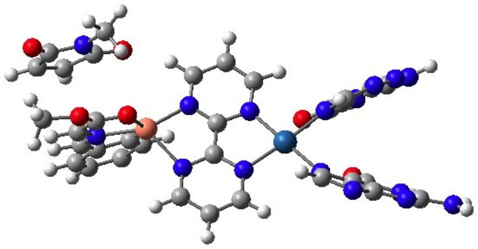

Center Number	Atomic Number	Atomic Type	Coordinates (Angstroms) X	Y	Z
1	7	0	-5.235566	-3.636416	-0.014596
2	6	0	-3.927964	-3.183214	-0.137262
3	7	0	-3.836665	-1.917273	0.299828
4	6	0	-5.136691	-1.530379	0.697654
5	6	0	-5.654254	-0.312121	1.280728
6	7	0	-7.083892	-0.396910	1.438566
7	6	0	-7.892508	-1.449041	1.037460
8	7	0	-9.242845	-1.308613	1.124631
9	7	0	-7.385406	-2.586228	0.549418
10	6	0	-6.033432	-2.584752	0.431909
11	1	0	-3.112403	-3.764587	-0.535113
12	1	0	-7.488183	0.422119	1.883163
13	1	0	-9.694003	-0.472471	1.466654
14	1	0	-9.813095	-2.098207	0.853255
15	7	0	-5.388247	0.634204	-2.613826
16	6	0	-4.350901	-0.196262	-2.234631
17	7	0	-3.605122	0.387262	-1.287236
18	6	0	-4.149999	1.660543	-1.068959
19	6	0	-3.747875	2.734137	-0.187974
20	8	0	-2.734303	2.836836	0.526821
21	7	0	-4.700459	3.811329	-0.254670
22	6	0	-5.825534	3.856967	-1.063277
23	7	0	-6.635704	4.950622	-0.999707
24	7	0	-6.145757	2.869008	-1.908412
25	6	0	-5.286736	1.815579	-1.876119
26	1	0	-4.160604	-1.168716	-2.653865
27	1	0	-4.473784	4.585280	0.363391
28	1	0	-6.414162	5.773621	-0.459176
29	1	0	-7.434718	4.970486	-1.618790
30	6	0	0.497821	-1.149647	0.642262
31	6	0	-0.866182	-2.825710	1.602696
32	6	0	0.273621	-3.421303	2.140425
33	1	0	-1.856214	-3.230427	1.775192
34	6	0	1.539846	-2.797203	1.904628
35	1	0	0.208501	-4.328076	2.725266
36	6	0	0.572819	-0.017781	-0.185276
37	6	0	-0.595457	1.559977	-1.495423
38	6	0	0.617126	2.167302	-1.821109
39	1	0	-1.538339	1.932383	-1.867638
40	6	0	1.818579	1.619213	-1.291070
41	1	0	0.649403	3.034559	-2.467507
42	1	0	2.796658	2.027898	-1.521812
43	7	0	-0.633654	0.462213	-0.693879
44	7	0	1.794589	0.545318	-0.481152
45	7	0	-0.780585	-1.699508	0.838166
46	7	0	1.645667	-1.680561	1.181944
47	7	0	5.257814	-0.476486	0.982630
48	6	0	5.988208	-1.500150	0.396587
49	6	0	5.346208	-2.282049	-0.595378
50	8	0	4.127169	-2.076622	-0.973744
51	6	0	5.814625	0.316849	1.886588
52	6	0	5.202830	1.457196	2.565132
53	6	0	3.947757	2.025157	2.219522
54	6	0	5.937077	2.060657	3.622346
55	6	0	3.455800	3.145022	2.905123
56	1	0	3.374558	1.598943	1.402014
57	6	0	5.438181	3.174734	4.311906
58	1	0	6.901757	1.641991	3.903301
59	6	0	4.191482	3.727234	3.957479

60	1	0	2.500918	3.576345	2.614981
61	1	0	6.023357	3.608593	5.117365
62	8	0	5.995873	-3.328765	-1.232538
63	6	0	7.420995	-3.575902	-0.987146
64	1	0	7.663253	-4.448529	-1.595998
65	1	0	8.022321	-2.721653	-1.313615
66	1	0	7.607148	-3.800273	0.071048
67	8	0	-5.066739	0.732759	1.628895
68	1	0	7.008949	-1.682988	0.715610
69	1	0	6.847933	0.105982	2.186005
70	6	0	8.019827	1.097084	-0.843087
71	6	0	6.984902	1.920052	-0.553394
72	1	0	8.983036	1.014834	-0.361935
73	1	0	6.884619	2.658435	0.229331
74	7	0	6.326849	0.640400	-2.383911
75	6	0	5.544714	0.020571	-3.457425
76	1	0	5.071590	0.797114	-4.066271
77	1	0	6.230957	-0.562439	-4.076610
78	1	0	4.782270	-0.647109	-3.042103
79	6	0	7.634008	0.235916	-2.008328
80	8	0	8.302902	-0.647446	-2.576978
81	6	0	5.878718	1.656276	-1.535934
82	8	0	4.768017	2.227175	-1.609783
83	78	0	-2.229677	-0.668116	-0.159313
84	29	0	3.328743	-0.578035	0.269016
85	1	0	2.455625	-3.214934	2.308430
86	1	0	3.807789	4.578170	4.480535

exo-RC

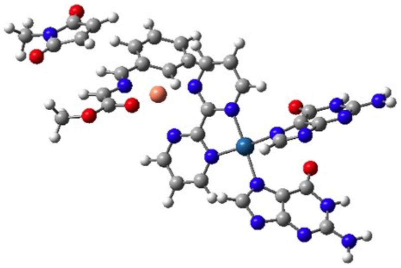

Center Number	Atomic Number	Atomic Type	Coordinates (Angstroms) X	Y	Z
1	7	0	-5.240437	-3.737132	0.356522
2	6	0	-3.998551	-3.170209	0.099330
3	7	0	-4.014093	-1.862270	0.401461
4	6	0	-5.325693	-1.564248	0.842011
5	6	0	-5.944415	-0.346793	1.318504
6	7	0	-7.337423	-0.563624	1.610463
7	6	0	-8.039636	-1.744729	1.424265
8	7	0	-9.376159	-1.757570	1.673935
9	7	0	-7.442340	-2.863985	1.003833
10	6	0	-6.115340	-2.728289	0.757261
11	1	0	-3.149848	-3.710024	-0.287613
12	1	0	-7.802777	0.256917	1.990806
13	1	0	-9.899169	-0.931710	1.925957
14	1	0	-9.860928	-2.640950	1.589686
15	7	0	-5.749560	0.399394	-2.694270
16	6	0	-4.652299	-0.323405	-2.263619
17	7	0	-3.968536	0.360996	-1.338349
18	6	0	-4.620462	1.592249	-1.182942
19	6	0	-4.321662	2.722309	-0.330652
20	8	0	-3.321862	2.934755	0.380035
21	7	0	-5.368925	3.701967	-0.421243
22	6	0	-6.488611	3.619824	-1.236498

23	7	0	-7.390137	4.641118	-1.190131
24	7	0	-6.710461	2.589169	-2.062813
25	6	0	-5.761687	1.615101	-2.000512
26	1	0	-4.382731	-1.298662	-2.630776
27	1	0	-5.229336	4.506236	0.186817
28	1	0	-7.235527	5.481598	-0.652884
29	1	0	-8.190826	4.592353	-1.805078
30	6	0	0.260515	-0.790769	0.600325
31	6	0	-0.956285	-2.538390	1.621792
32	6	0	0.232606	-3.032440	2.155466
33	1	0	-1.908756	-3.010218	1.827760
34	6	0	1.439976	-2.312543	1.896442
35	1	0	0.247888	-3.928701	2.758469
36	1	0	2.389799	-2.638071	2.305932
37	6	0	0.243785	0.311136	-0.270267
38	6	0	-1.060165	1.746251	-1.616848
39	6	0	0.093479	2.455298	-1.958848
40	1	0	-2.033973	2.010911	-2.000195
41	6	0	1.335734	2.019028	-1.424398
42	1	0	0.049558	3.314223	-2.613764
43	1	0	2.261279	2.528655	-1.664352
44	7	0	-1.001458	0.675526	-0.784532
45	7	0	1.417486	0.958469	-0.604884
46	7	0	-0.969567	-1.429265	0.827721
47	7	0	1.445944	-1.209477	1.149711
48	7	0	4.973318	-0.273514	0.511549
49	6	0	5.446703	-1.481641	0.019304
50	6	0	4.579153	-2.234134	-0.817737
51	8	0	3.411293	-1.815681	-1.173755
52	6	0	5.744917	0.547952	1.207695
53	6	0	5.339991	1.830687	1.792003
54	6	0	3.990810	2.249725	1.939281
55	6	0	6.362154	2.702545	2.256162
56	6	0	3.680703	3.487489	2.522523
57	1	0	3.185909	1.591493	1.622047
58	6	0	6.047600	3.938744	2.840199
59	1	0	7.404461	2.415910	2.136923
60	6	0	4.705564	4.341383	2.977579
61	1	0	2.640776	3.783924	2.638488
62	1	0	6.846723	4.587921	3.185939
63	1	0	4.463206	5.294220	3.438691
64	8	0	4.931968	-3.482829	-1.306131
65	6	0	6.206282	-4.106980	-0.929441
66	8	0	-5.462495	0.792202	1.483869
67	1	0	6.431310	-1.833488	0.304614
68	1	0	6.780203	0.251652	1.399471
69	6	0	7.422184	-0.149680	-1.961773
70	6	0	7.537656	1.063216	-1.363605
71	7	0	9.396961	-0.193140	-0.723014
72	6	0	10.677407	-0.596212	-0.139507
73	1	0	11.131080	0.288302	0.316068
74	1	0	10.539816	-1.364620	0.628946
75	1	0	11.338052	-0.992903	-0.916963
76	6	0	8.606355	-0.989887	-1.574667
77	8	0	8.874545	-2.161748	-1.913295
78	6	0	8.797101	1.078981	-0.548882
79	8	0	9.265492	1.993044	0.158250
80	1	0	6.655209	-0.515187	-2.627648
81	1	0	6.880328	1.917202	-1.427115
82	29	0	3.022241	-0.092453	-0.013471
83	78	0	-2.510043	-0.537231	-0.175243
84	1	0	6.027855	-5.106607	-0.592086
85	1	0	6.857790	-4.127856	-1.777970
86	1	0	6.661958	-3.541375	-0.143725

----

endo-P

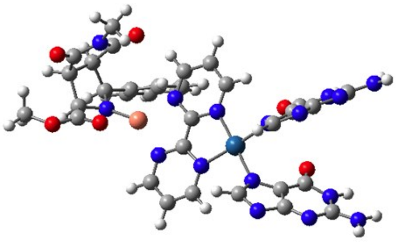

Center Number	Atomic Number	Atomic Type	Coordinates (Angstroms) X	Y	Z
1	7	0	-5.118480	-3.672855	-0.045110
2	6	0	-3.818563	-3.192991	-0.161359
3	7	0	-3.752407	-1.928301	0.284464
4	6	0	-5.061047	-1.570048	0.682016
5	6	0	-5.601284	-0.371122	1.282814
6	7	0	-7.029000	-0.489262	1.446729
7	6	0	-7.816657	-1.553119	1.036523
8	7	0	-9.169964	-1.443747	1.137834
9	7	0	-7.289112	-2.671423	0.528797
10	6	0	-5.936710	-2.640094	0.406538
11	1	0	-2.989468	-3.754926	-0.559125
12	1	0	-7.448257	0.315788	1.902515
13	1	0	-9.635665	-0.622627	1.496283
14	1	0	-9.725078	-2.241211	0.858893
15	7	0	-5.348737	0.621759	-2.585128
16	6	0	-4.283128	-0.187072	-2.234044
17	7	0	-3.542569	0.398068	-1.282924
18	6	0	-4.120569	1.652041	-1.034756
19	6	0	-3.744699	2.718141	-0.133127
20	8	0	-2.729014	2.841594	0.575013
21	7	0	-4.733517	3.765185	-0.165018
22	6	0	-5.866475	3.794215	-0.961980
23	7	0	-6.710006	4.861387	-0.865954
24	7	0	-6.163585	2.816884	-1.826343
25	6	0	-5.271013	1.789777	-1.825470
26	1	0	-4.073194	-1.146942	-2.672658
27	1	0	-4.525980	4.529767	0.470965
28	1	0	-6.505405	5.680204	-0.312993
29	1	0	-7.514124	4.870634	-1.478492
30	6	0	0.560522	-1.116975	0.672081
31	6	0	-0.796565	-2.827255	1.589392
32	6	0	0.340771	-3.417224	2.144758
33	6	0	1.601012	-2.764308	1.957496
34	1	0	0.280582	-4.340931	2.700811
35	1	0	2.510040	-3.161388	2.397476
36	6	0	0.642087	0.006758	-0.131587
37	6	0	-0.523616	1.632071	-1.395224
38	6	0	0.686254	2.279871	-1.656572
39	6	0	1.885821	1.712484	-1.129770
40	1	0	0.715685	3.197145	-2.228176
41	1	0	2.851505	2.171522	-1.320422
42	7	0	-0.571042	0.494420	-0.661780
43	7	0	1.866692	0.614461	-0.366620
44	7	0	-0.721067	-1.691900	0.847273
45	7	0	1.708914	-1.652631	1.236096
46	7	0	5.251615	-0.396928	0.870371
47	6	0	6.182516	-1.282857	0.177325
48	6	0	5.438074	-2.413409	-0.547349
49	8	0	4.204563	-2.383495	-0.713102
50	6	0	6.087073	0.776472	1.197383
51	6	0	5.432463	1.881462	2.009299
52	6	0	4.149968	2.399363	1.735161
53	6	0	6.128920	2.388071	3.128029
54	6	0	3.584694	3.394009	2.550031
55	1	0	3.593155	2.034177	0.883922
56	6	0	5.571473	3.387833	3.944238
57	1	0	7.110818	1.989474	3.374793
58	6	0	4.291503	3.896006	3.658518
59	1	0	2.596953	3.783195	2.316736
60	1	0	6.133796	3.759135	4.795756

61	1	0	3.852143	4.669301	4.282478
62	8	0	6.120531	-3.529653	-0.946036
63	6	0	7.591548	-3.583844	-1.030254
64	1	0	7.817632	-4.629944	-1.236370
65	1	0	7.921869	-2.951333	-1.858418
66	1	0	8.055863	-3.283980	-0.083990
67	8	0	-5.036952	0.682511	1.643675
68	1	0	6.901682	-1.749855	0.878082
69	1	0	6.957830	0.446892	1.801470
70	6	0	7.085373	-0.314409	-0.757176
71	6	0	6.731055	1.097617	-0.212228
72	1	0	8.142251	-0.577001	-0.694932
73	1	0	7.592190	1.771384	-0.136535
74	7	0	5.785077	0.784202	-2.384209
75	6	0	5.047645	1.026228	-3.625794
76	1	0	4.899581	2.100398	-3.743927
77	1	0	5.627158	0.626080	-4.460672
78	1	0	4.073489	0.526172	-3.600443
79	6	0	6.623036	-0.320236	-2.200879
80	8	0	6.891960	-1.159936	-3.083793
81	6	0	5.758124	1.644755	-1.267562
82	8	0	5.064766	2.678949	-1.229951
83	78	0	-2.158659	-0.657480	-0.159336
84	29	0	3.362006	-0.583738	0.632442
85	1	0	-1.751772	-3.280983	1.752521
86	1	0	-1.432179	2.043452	-1.782753

exo-P

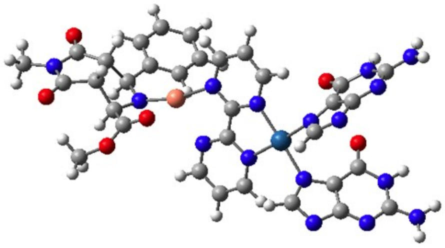

Center Number	Atomic Number	Atomic Type	Coordinates (Angstroms) X	Y	Z
1	7	0	-5.194809	-3.732651	0.485084
2	6	0	-3.958988	-3.170017	0.189262
3	7	0	-3.975402	-1.851845	0.441948
4	6	0	-5.283385	-1.542228	0.887744
5	6	0	-5.903960	-0.310635	1.324277
6	7	0	-7.290224	-0.524809	1.649333
7	6	0	-7.988326	-1.715191	1.518508
8	7	0	-9.320260	-1.725502	1.792604
9	7	0	-7.391467	-2.845361	1.128699
10	6	0	-6.068518	-2.712189	0.858358
11	1	0	-3.112829	-3.721810	-0.185418
12	1	0	-7.754319	0.307412	2.004705
13	1	0	-9.842869	-0.893094	2.022029
14	1	0	-9.801879	-2.613542	1.749342
15	7	0	-5.707521	0.350501	-2.697020
16	6	0	-4.614308	-0.365128	-2.243820
17	7	0	-3.925731	0.342189	-1.339937
18	6	0	-4.570382	1.581546	-1.221561
19	6	0	-4.271807	2.730120	-0.394426
20	8	0	-3.279733	2.951695	0.324880
21	7	0	-5.308919	3.716402	-0.521838
22	6	0	-6.426465	3.617974	-1.337779
23	7	0	-7.320088	4.647522	-1.325334
24	7	0	-6.654357	2.564381	-2.132440
25	6	0	-5.711664	1.586539	-2.040134
26	1	0	-4.350533	-1.352835	-2.581169
27	1	0	-5.167126	4.535596	0.065356

28	1	0	-7.158030	5.504526	-0.817120
29	1	0	-8.121108	4.584913	-1.938382
30	6	0	0.318767	-0.836554	0.566973
31	6	0	-0.928128	-2.524597	1.661254
32	6	0	0.251997	-3.026580	2.203153
33	1	0	-1.888941	-2.965248	1.896129
34	6	0	1.474159	-2.344573	1.899515
35	1	0	0.252336	-3.895889	2.843974
36	1	0	2.421330	-2.670182	2.316094
37	6	0	0.303591	0.277634	-0.285494
38	6	0	-1.023869	1.770306	-1.551559
39	6	0	0.116024	2.522419	-1.845267
40	1	0	-2.000572	2.041815	-1.920511
41	6	0	1.360970	2.082386	-1.325476
42	1	0	0.058252	3.420099	-2.444892
43	1	0	2.275202	2.631135	-1.517019
44	7	0	-0.949410	0.656667	-0.781935
45	7	0	1.463902	0.974642	-0.574042
46	7	0	-0.927031	-1.443117	0.828669
47	7	0	1.498468	-1.279932	1.105330
48	7	0	4.950567	-0.316379	0.485453
49	6	0	5.644704	-1.319982	-0.318587
50	6	0	4.641177	-2.142225	-1.136087
51	8	0	3.551406	-1.659432	-1.507969
52	6	0	6.018067	0.575934	0.944825
53	6	0	5.559510	1.847018	1.659214
54	6	0	4.241023	1.999250	2.139010
55	6	0	6.486503	2.884914	1.913066
56	6	0	3.845161	3.171462	2.811733
57	1	0	3.529519	1.189400	2.010688
58	6	0	6.093924	4.050933	2.592137
59	1	0	7.516684	2.783216	1.584509
60	6	0	4.768150	4.207753	3.035673
61	1	0	2.823902	3.266756	3.174189
62	1	0	6.826313	4.829312	2.784546
63	1	0	4.466518	5.113703	3.553325
64	8	0	4.888959	-3.448640	-1.454506
65	6	0	6.110137	-4.185191	-1.061709
66	1	0	5.954672	-5.186185	-1.466998
67	1	0	6.995214	-3.722818	-1.503494
68	1	0	6.191703	-4.234555	0.029918
69	8	0	-5.430723	0.838537	1.433808
70	1	0	6.257040	-2.006050	0.296095
71	1	0	6.694978	0.037943	1.645755
72	6	0	6.658191	-0.464933	-1.222312
73	6	0	6.881965	0.822968	-0.385803
74	7	0	8.982370	-0.226373	-0.807778
75	6	0	10.426565	-0.484569	-0.798778
76	1	0	10.932568	0.445237	-0.527818
77	1	0	10.679600	-1.260451	-0.067490
78	1	0	10.745151	-0.819905	-1.789377
79	6	0	8.045274	-1.062190	-1.424152
80	8	0	8.343082	-2.121445	-2.017581
81	6	0	8.395195	0.916491	-0.217295
82	8	0	9.066539	1.807515	0.335895
83	1	0	6.200983	-0.270575	-2.196070
84	1	0	6.505439	1.723859	-0.875598
85	29	0	3.142408	0.106850	-0.016040
86	78	0	-2.464531	-0.546459	-0.168214

endo-TS

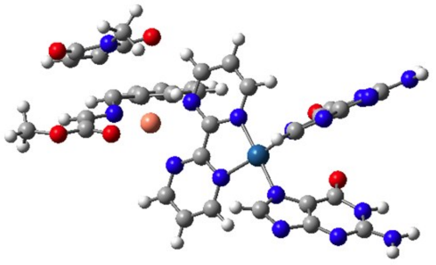

Center Number	Atomic Number	Atomic Type	Coordinates (Angstroms) X	Y	Z
1	7	0	-5.090349	-3.665246	-0.114357
2	6	0	-3.797219	-3.171730	-0.221366
3	7	0	-3.742600	-1.921521	0.264651
4	6	0	-5.050552	-1.588210	0.679615
5	6	0	-5.595112	-0.408921	1.314635
6	7	0	-7.017953	-0.546149	1.493345
7	6	0	-7.799541	-1.608178	1.063171
8	7	0	-9.151121	-1.517931	1.185820
9	7	0	-7.266329	-2.706907	0.517188
10	6	0	-5.917415	-2.658220	0.379092
11	1	0	-2.966271	-3.714194	-0.642180
12	1	0	-7.441355	0.239394	1.978834
13	1	0	-9.622446	-0.711418	1.569664
14	1	0	-9.700927	-2.312944	0.889140
15	7	0	-5.263467	0.663937	-2.649751
16	6	0	-4.211386	-0.134344	-2.245769
17	7	0	-3.544499	0.442562	-1.237112
18	6	0	-4.157838	1.681404	-1.002190
19	6	0	-3.857980	2.729544	-0.052872
20	8	0	-2.897376	2.835143	0.730744
21	7	0	-4.851054	3.768358	-0.131900
22	6	0	-5.907112	3.819657	-1.029461
23	7	0	-6.741351	4.896565	-0.995999
24	7	0	-6.135845	2.854118	-1.928695
25	6	0	-5.251411	1.823046	-1.870570
26	1	0	-3.962048	-1.084094	-2.687232
27	1	0	-4.717864	4.507022	0.553082
28	1	0	-6.570999	5.710579	-0.423991
29	1	0	-7.489837	4.925063	-1.675376
30	6	0	0.558272	-1.113176	0.667395
31	6	0	-0.789706	-2.835222	1.561035
32	6	0	0.355179	-3.443463	2.074782
33	1	0	-1.776525	-3.252659	1.719285
34	6	0	1.613976	-2.795953	1.873213
35	1	0	0.298650	-4.374977	2.619837
36	1	0	2.533493	-3.215078	2.266649
37	6	0	0.627192	0.055865	-0.109723
38	6	0	-0.555844	1.737557	-1.264246
39	6	0	0.651959	2.381118	-1.535781
40	1	0	-1.502654	2.140716	-1.593027
41	6	0	1.862160	1.788023	-1.073901
42	1	0	0.672929	3.311226	-2.088236
43	1	0	2.837444	2.238279	-1.252011
44	7	0	-0.585452	0.569749	-0.568225
45	7	0	1.845098	0.637366	-0.377809
46	7	0	-0.712766	-1.679856	0.840931
47	7	0	1.706583	-1.648593	1.199187
48	7	0	5.336477	-0.484482	0.773126
49	6	0	6.031105	-1.508696	0.237967
50	6	0	5.290446	-2.470820	-0.581929
51	8	0	4.037009	-2.375033	-0.742676
52	6	0	6.106287	0.544036	1.310775
53	6	0	5.506729	1.615794	2.161101
54	6	0	4.192980	2.108779	1.994456
55	6	0	6.315613	2.200949	3.164005
56	6	0	3.706323	3.148104	2.800629
57	1	0	3.559791	1.695536	1.219490
58	6	0	5.831006	3.243684	3.972374
59	1	0	7.328418	1.833634	3.318142
60	6	0	4.519924	3.723535	3.794383

61	1	0	2.698517	3.524507	2.644983
62	1	0	6.474332	3.679389	4.731434
63	1	0	4.138648	4.536350	4.405632
64	8	0	5.931407	-3.521423	-1.176083
65	8	0	-5.030432	0.639307	1.689509
66	1	0	7.054466	-1.711452	0.533869
67	1	0	7.085289	0.222564	1.690614
68	6	0	7.560861	0.280521	-1.039649
69	6	0	6.879788	1.314088	-0.328510
70	1	0	8.538474	-0.122452	-0.824897
71	1	0	7.391923	2.127131	0.180897
72	7	0	5.707833	0.846061	-2.301123
73	6	0	4.805204	0.930909	-3.442258
74	1	0	4.679318	1.977075	-3.738046
75	1	0	5.258884	0.367895	-4.261385
76	1	0	3.822646	0.501285	-3.217141
77	6	0	6.851405	-0.041400	-2.258054
78	8	0	7.098128	-0.862919	-3.179888
79	6	0	5.704172	1.705056	-1.212948
80	8	0	4.857874	2.620999	-1.034328
81	78	0	-2.168225	-0.616823	-0.115364
82	29	0	3.367877	-0.569478	0.368497
83	6	0	7.320994	-3.858237	-1.153605
84	1	0	7.484636	-4.736672	-1.742225
85	1	0	7.891077	-3.047449	-1.556753
86	1	0	7.627030	-4.042611	-0.145018

exo-TS

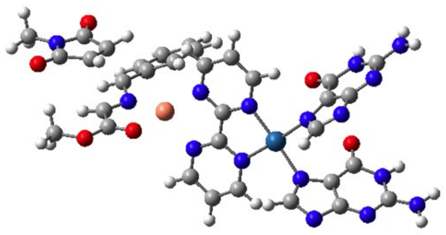

Center Number	Atomic Number	Atomic Type	Coordinates (Angstroms) X	Y	Z
1	7	0	-5.499657	-3.589472	0.268431
2	6	0	-4.193418	-3.138933	0.118090
3	7	0	-4.108253	-1.840664	0.450936
4	6	0	-5.413595	-1.431349	0.808278
5	6	0	-5.937496	-0.176222	1.296132
6	7	0	-7.365509	-0.256347	1.466588
7	6	0	-8.169432	-1.337371	1.137121
8	7	0	-9.519450	-1.193834	1.219045
9	7	0	-7.656797	-2.503559	0.730449
10	6	0	-6.303891	-2.506396	0.622313
11	1	0	-3.371786	-3.746689	-0.222750
12	1	0	-7.778503	0.576849	1.874920
13	1	0	-9.969458	-0.336322	1.506057
14	1	0	-10.091410	-1.998069	0.999600
15	7	0	-5.713394	0.663241	-2.509190
16	6	0	-4.709159	-0.193701	-2.107254
17	7	0	-3.892640	0.412326	-1.237043
18	6	0	-4.356430	1.728130	-1.092509
19	6	0	-3.870388	2.829662	-0.291088
20	8	0	-2.837484	2.909707	0.399579
21	7	0	-4.754107	3.958765	-0.408895
22	6	0	-5.899838	4.016314	-1.188767
23	7	0	-6.636009	5.162005	-1.181071
24	7	0	-6.308997	2.991138	-1.948731
25	6	0	-5.514237	1.891417	-1.868736
26	1	0	-4.588116	-1.202547	-2.460014

27	1	0	-4.468801	4.758455	0.152039
28	1	0	-6.356148	5.997178	-0.687966
29	1	0	-7.453521	5.193786	-1.774653
30	6	0	0.255119	-1.158639	0.537155
31	6	0	-1.091898	-2.720463	1.685943
32	6	0	0.062316	-3.307271	2.203867
33	1	0	-2.080094	-3.079668	1.944834
34	6	0	1.326439	-2.739967	1.858305
35	1	0	0.009938	-4.158887	2.865203
36	1	0	2.252173	-3.150750	2.245310
37	6	0	0.317148	-0.092866	-0.374395
38	6	0	-0.874157	1.414409	-1.745029
39	6	0	0.337057	1.936008	-2.202214
40	1	0	-1.823952	1.792544	-2.093621
41	6	0	1.544672	1.382534	-1.691052
42	1	0	0.359371	2.737256	-2.927505
43	1	0	2.512100	1.738204	-2.023937
44	7	0	-0.902558	0.395529	-0.846429
45	7	0	1.538660	0.393653	-0.785972
46	7	0	-1.020973	-1.657326	0.836006
47	7	0	1.416807	-1.676844	1.056121
48	7	0	5.031437	-0.560352	0.510720
49	6	0	5.740965	-1.541640	-0.103428
50	6	0	4.991383	-2.520025	-0.892034
51	8	0	3.734507	-2.419446	-1.051533
52	6	0	5.806041	0.462116	1.040453
53	6	0	5.206961	1.588650	1.813674
54	6	0	4.011263	2.239029	1.427739
55	6	0	5.905903	2.085736	2.936840
56	6	0	3.531382	3.346421	2.147561
57	1	0	3.465878	1.885729	0.556146
58	6	0	5.423895	3.186316	3.664015
59	1	0	6.840225	1.616018	3.230933
60	6	0	4.233313	3.824245	3.271545
61	1	0	2.620040	3.848033	1.831226
62	1	0	5.979728	3.550096	4.523489
63	8	0	5.614285	-3.585315	-1.479321
64	6	0	7.083533	-3.756670	-1.432323
65	1	0	7.271575	-4.634699	-2.051049
66	1	0	7.596342	-2.885807	-1.850873
67	1	0	7.409961	-3.951859	-0.403763
68	8	0	-5.345866	0.889856	1.559201
69	1	0	6.765917	-1.755679	0.185003
70	1	0	6.763244	0.135169	1.465992
71	6	0	6.777825	0.166227	-1.581727
72	6	0	6.581339	1.214673	-0.629786
73	1	0	6.102644	-0.131536	-2.369307
74	1	0	5.849064	2.003895	-0.762304
75	7	0	8.870232	0.721300	-0.733569
76	6	0	10.315755	0.742658	-0.531212
77	1	0	10.551453	0.846653	0.531369
78	1	0	10.724261	-0.194591	-0.917154
79	1	0	10.776063	1.576485	-1.071175
80	6	0	8.201547	-0.166409	-1.634367
81	8	0	8.805977	-1.052424	-2.292768
82	6	0	7.964671	1.636468	-0.174062
83	8	0	8.278365	2.576233	0.586446
84	78	0	-2.499386	-0.639849	-0.139099
85	29	0	3.074561	-0.678964	0.088732
86	1	0	3.864263	4.664580	3.821581

endo-RC-(Cu-bipym)

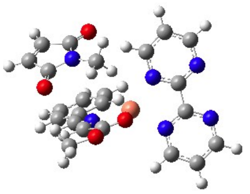

Center Number	Atomic Number	Atomic Type	Coordinates (Angstroms) X	Y	Z
1	6	0	3.622808	-0.718940	0.120685
2	6	0	5.438528	-1.374453	-1.147039
3	6	0	4.634024	-1.432349	-2.300652
4	1	0	6.496762	-1.612961	-1.174161
5	6	0	3.280054	-1.092503	-2.155474
6	1	0	5.039433	-1.725497	-3.261050
7	1	0	2.594177	-1.116228	-2.993907
8	6	0	3.016609	-0.366929	1.438471
9	6	0	3.223417	-0.007365	3.710842
10	6	0	1.837296	0.199340	3.820983
11	1	0	3.883464	0.050146	4.569427
12	6	0	1.073991	0.121693	2.643857
13	1	0	1.366141	0.413999	4.772209
14	1	0	0.000990	0.279589	2.659551
15	7	0	3.811290	-0.293555	2.522818
16	7	0	1.661655	-0.155966	1.447471
17	7	0	4.934447	-1.023866	0.060898
18	7	0	2.772280	-0.727411	-0.950625
19	7	0	-0.879106	0.214709	-1.446018
20	6	0	-1.637297	-0.847581	-1.838309
21	6	0	-1.175249	-2.187018	-1.476905
22	8	0	-0.118474	-2.323462	-0.793742
23	6	0	-1.299281	1.448643	-1.813489
24	6	0	-0.690149	2.708914	-1.483307
25	6	0	0.467572	2.853797	-0.663132
26	6	0	-1.306785	3.885571	-2.009885
27	6	0	0.975990	4.124666	-0.382178
28	1	0	0.951474	1.973308	-0.249929
29	6	0	-0.790199	5.153423	-1.727339
30	1	0	-2.188907	3.788549	-2.638425
31	6	0	0.352691	5.278237	-0.908869
32	1	0	1.855762	4.230304	0.244205
33	1	0	-1.266892	6.038789	-2.135244
34	1	0	0.755147	6.261268	-0.682395
35	8	0	-1.854333	-3.293384	-1.867371
36	1	0	-2.557280	-0.697162	-2.396146
37	1	0	-2.199672	1.505579	-2.434642
38	6	0	-4.929157	0.463610	0.933582
39	6	0	-4.183539	1.161989	1.818137
40	1	0	-5.827108	0.757987	0.408936
41	1	0	-4.326070	2.164781	2.194926
42	7	0	-3.189521	-0.942642	1.611341
43	6	0	-2.338366	-2.120475	1.826235
44	1	0	-2.328106	-2.386036	2.886803
45	1	0	-2.757896	-2.949564	1.252648
46	1	0	-1.314936	-1.940474	1.486936
47	6	0	-4.301338	-0.892788	0.743671
48	8	0	-4.640257	-1.792990	-0.046177
49	6	0	-3.028896	0.297980	2.254559
50	8	0	-2.078657	0.601802	3.001376
51	6	0	-3.131031	-3.225409	-2.615476
52	1	0	-3.369597	-4.265741	-2.828718
53	1	0	-3.903419	-2.779155	-1.983908
54	1	0	-2.997355	-2.678300	-3.555035
55	29	0	0.799316	-0.377245	-0.412061

exo-RC-(Cu-bipym)

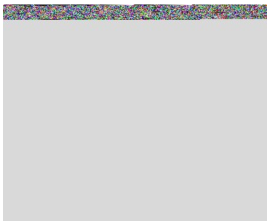

Center Number	Atomic Number	Atomic Type	Coordinates (Angstroms) X	Y	Z
1	6	0	4.237324	-0.771863	0.350371
2	6	0	6.111374	-1.304049	-0.888293
3	6	0	5.375504	-1.186026	-2.082203
4	1	0	7.162908	-1.569813	-0.888518
5	6	0	4.019422	-0.842532	-1.969951
6	1	0	5.832395	-1.353632	-3.049421
7	1	0	3.387036	-0.731720	-2.842869
8	6	0	3.560408	-0.574903	1.665613
9	6	0	3.627975	-0.479486	3.973550
10	6	0	2.236691	-0.269953	4.023284
11	1	0	4.231381	-0.528146	4.873808
12	6	0	1.553828	-0.215087	2.799432
13	1	0	1.711546	-0.157853	4.963462
14	1	0	0.483535	-0.055405	2.750787
15	7	0	4.286964	-0.633911	2.799543
16	7	0	2.208406	-0.358771	1.618464
17	7	0	5.545144	-1.095110	0.325439
18	7	0	3.445567	-0.634370	-0.757498
19	7	0	-0.221985	0.270887	-1.294367
20	6	0	-0.989675	-0.758960	-1.744208
21	6	0	-0.539128	-2.121243	-1.446006
22	8	0	0.521181	-2.302308	-0.780317
23	6	0	-0.606995	1.528308	-1.615509
24	6	0	-0.018297	2.758468	-1.152123
25	6	0	0.996127	2.838381	-0.153047
26	6	0	-0.509762	3.971018	-1.723363
27	6	0	1.494318	4.082150	0.246083
28	1	0	1.378456	1.929707	0.303519
29	6	0	0.005318	5.210430	-1.329890
30	1	0	-1.290380	3.926529	-2.479238
31	6	0	1.009371	5.271631	-0.341569
32	1	0	2.258943	4.141198	1.013607
33	1	0	-0.369395	6.123107	-1.781230
34	8	0	-1.230600	-3.200435	-1.888345
35	6	0	-2.494493	-3.087426	-2.651824
36	1	0	-2.844893	-4.115368	-2.737049
37	1	0	-3.217062	-2.478686	-2.102964
38	1	0	-2.296069	-2.679336	-3.648861
39	1	0	-1.920885	-0.570269	-2.268429
40	1	0	-1.454688	1.632598	-2.298922
41	6	0	-4.355803	-0.832078	1.810833
42	6	0	-5.437131	-0.477417	2.537928
43	1	0	-3.389895	-1.182176	2.145631
44	1	0	-5.568491	-0.454038	3.610546
45	7	0	-6.013732	-0.264666	0.276657
46	6	0	-6.778695	-0.045324	-0.954601
47	1	0	-6.462138	0.872048	-1.460873
48	1	0	-6.647962	-0.893166	-1.632910
49	1	0	-7.833011	0.046111	-0.683461
50	6	0	-4.688516	-0.696499	0.348185
51	8	0	-3.936608	-0.925542	-0.627191
52	6	0	-6.545106	-0.099628	1.588298
53	8	0	-7.695023	0.275832	1.850662
54	1	0	1.406951	6.231900	-0.027813
55	29	0	1.442525	-0.389305	-0.300720

endo-P-(Cu-bipym)

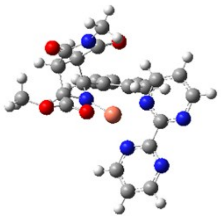

Center Number	Atomic Number	Atomic Type	Coordinates (Angstroms) X	Y	Z
1	6	0	3.569470	-0.519436	-0.094880
2	6	0	5.273866	-1.364265	-1.400651
3	6	0	4.391834	-1.519978	-2.486774
4	1	0	6.321431	-1.636398	-1.470966
5	6	0	3.055173	-1.141382	-2.287719
6	1	0	4.726741	-1.915970	-3.437449
7	1	0	2.311627	-1.230310	-3.070327
8	6	0	3.060115	0.047141	1.186608
9	6	0	3.411043	0.825516	3.330127
10	6	0	2.045161	1.135666	3.459346
11	1	0	4.120044	1.014352	4.128584
12	6	0	1.209061	0.855726	2.366119
13	1	0	1.646633	1.578016	4.363881
14	1	0	0.147967	1.082535	2.388618
15	7	0	3.918088	0.283303	2.194500
16	7	0	1.716708	0.308675	1.228986
17	7	0	4.864071	-0.864511	-0.208239
18	7	0	2.645225	-0.641466	-1.095309
19	7	0	-1.022345	-0.075157	-1.132864
20	6	0	-1.869427	-1.293248	-1.201204
21	6	0	-1.058083	-2.527100	-0.777926
22	8	0	0.070108	-2.382258	-0.258076
23	6	0	-2.009841	1.037200	-1.196084
24	6	0	-1.495895	2.464808	-1.198020
25	6	0	-0.393375	2.917664	-0.446819
26	6	0	-2.178582	3.389461	-2.019207
27	6	0	0.023027	4.257732	-0.525966
28	1	0	0.134734	2.240559	0.214635
29	6	0	-1.776360	4.733517	-2.088116
30	1	0	-3.030861	3.061310	-2.610784
31	6	0	-0.665405	5.171411	-1.344882
32	1	0	0.874900	4.595015	0.057311
33	1	0	-2.319828	5.427868	-2.721465
34	1	0	-0.344104	6.207478	-1.400735
35	8	0	-1.510780	-3.766759	-1.045112
36	1	0	-2.187963	-1.443448	-2.244828
37	1	0	-2.568976	0.921840	-2.141722
38	6	0	-3.185487	-0.950437	-0.365032
39	6	0	-3.026182	0.572129	-0.095096
40	1	0	-4.082817	-1.220113	-0.922986
41	1	0	-3.967028	1.127332	-0.150096
42	7	0	-2.753988	-0.646975	1.939210
43	6	0	-2.533213	-0.939697	3.362875
44	1	0	-3.018118	-0.177517	3.978003
45	1	0	-2.960176	-1.921747	3.575725
46	1	0	-1.462415	-0.956547	3.590672
47	6	0	-3.217412	-1.595654	1.018624
48	8	0	-3.561665	-2.757603	1.293301
49	6	0	-2.507487	0.609283	1.353742
50	8	0	-1.963407	1.549505	1.958979
51	6	0	-2.863598	-4.061999	-1.571492
52	1	0	-2.824664	-5.117368	-1.834439
53	1	0	-3.591288	-3.887345	-0.777013
54	1	0	-3.075810	-3.465716	-2.465289
55	29	0	0.750042	-0.227529	-0.516947

exo-P-(Cu-bipym)

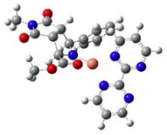

Center Number	Atomic Number	Atomic Type	Coordinates (Angstroms) X	Y	Z

1	6	0	3.872712	-0.711068	-0.442867
2	6	0	5.143992	-1.909544	-1.949860
3	6	0	3.987046	-2.276677	-2.663163
4	1	0	6.131631	-2.239523	-2.253760
5	6	0	2.758009	-1.800161	-2.182586
6	1	0	4.038096	-2.899844	-3.546919
7	1	0	1.821264	-2.035706	-2.672759
8	6	0	3.738000	0.121313	0.786109
9	6	0	4.660707	1.237713	2.583175
10	6	0	3.377591	1.569335	3.057786
11	1	0	5.561886	1.543664	3.104136
12	6	0	2.280598	1.133584	2.298479
13	1	0	3.237658	2.138875	3.968146
14	1	0	1.262266	1.347219	2.595497
15	7	0	4.839233	0.513824	1.450495
16	7	0	2.455585	0.415013	1.162214
17	7	0	5.087289	-1.127412	-0.842869
18	7	0	2.699863	-1.018962	-1.075360
19	7	0	-0.818929	-0.259336	-0.412535
20	6	0	-1.703646	-1.391608	-0.036557
21	6	0	-0.840488	-2.507795	0.584603
22	8	0	0.314209	-2.240498	0.989469
23	6	0	-1.721014	0.896667	-0.675494
24	6	0	-1.002758	2.224880	-0.799729
25	6	0	-0.302416	2.772504	0.296148
26	6	0	-1.035930	2.940502	-2.012110
27	6	0	0.356416	4.006707	0.182325
28	1	0	-0.296268	2.247137	1.248848
29	6	0	-0.381133	4.180012	-2.130540
30	1	0	-1.583002	2.540283	-2.862225
31	6	0	0.317755	4.716141	-1.033922
32	1	0	0.881881	4.422992	1.037176
33	1	0	-0.427115	4.727179	-3.067276
34	8	0	-1.269567	-3.782638	0.648082
35	6	0	-2.521732	-4.311575	0.043515
36	1	0	-2.481414	-5.377748	0.258340
37	1	0	-3.396668	-3.849174	0.501862
38	1	0	-2.509382	-4.149921	-1.040396
39	1	0	-2.200539	-1.788704	-0.936438
40	1	0	-2.261113	0.703490	-1.617785
41	6	0	-2.799816	-0.720639	0.886871
42	6	0	-2.744729	0.787279	0.516547
43	1	0	-2.583038	-0.907338	1.941984
44	1	0	-2.418259	1.421917	1.341820
45	7	0	-4.957168	-0.013079	0.163609
46	6	0	-6.381418	-0.039204	-0.205620
47	1	0	-6.496346	0.070188	-1.288827
48	1	0	-6.798951	-0.996678	0.111704
49	1	0	-6.903432	0.785359	0.285448
50	6	0	-4.250636	-1.134851	0.599959
51	8	0	-4.738265	-2.273140	0.728561
52	6	0	-4.172549	1.160709	0.122236
53	8	0	-4.599693	2.274757	-0.206011
54	1	0	0.815298	5.677124	-1.119863
55	29	0	1.034607	-0.360149	-0.115181

endo-TS-(Cu-bipym)

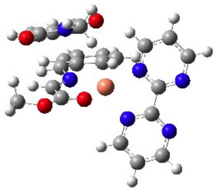

Center Number	Atomic Number	Atomic Type	Coordinates (Angstroms) X	Y	Z

1	6	0	3.556374	-0.564156	-0.274827
2	6	0	5.091734	-1.444402	-1.757437
3	6	0	4.074793	-1.667879	-2.704643
4	1	0	6.126620	-1.702510	-1.955734
5	6	0	2.771621	-1.287131	-2.347434
6	1	0	4.286161	-2.114184	-3.668637
7	1	0	1.933265	-1.433240	-3.017954
8	6	0	3.220988	0.010833	1.060621
9	6	0	3.873806	0.776009	3.140263
10	6	0	2.537404	1.056088	3.475164
11	1	0	4.690191	0.962831	3.829609
12	6	0	1.554140	0.773179	2.513032
13	1	0	2.267498	1.476566	4.435975
14	1	0	0.501961	0.975107	2.680981
15	7	0	4.215059	0.259737	1.933597
16	7	0	1.892900	0.243575	1.305279
17	7	0	4.833068	-0.900930	-0.543495
18	7	0	2.510780	-0.724272	-1.140776
19	7	0	-1.237454	-0.125856	-0.957272
20	6	0	-1.828187	-1.278677	-1.236842
21	6	0	-1.103818	-2.526769	-0.895238
22	8	0	0.039352	-2.452976	-0.376976
23	6	0	-2.016383	1.047739	-1.173608
24	6	0	-1.418203	2.391328	-1.264548
25	6	0	-0.203239	2.772661	-0.643589
26	6	0	-2.149800	3.367865	-1.993065
27	6	0	0.271545	4.085604	-0.769786
28	1	0	0.356308	2.057052	-0.049749
29	6	0	-1.676203	4.680077	-2.112655
30	1	0	-3.088952	3.093237	-2.468395
31	6	0	-0.459006	5.043605	-1.500890
32	1	0	1.203231	4.371760	-0.291762
33	1	0	-2.243898	5.413170	-2.677048
34	1	0	-0.088231	6.060635	-1.588594
35	8	0	-1.654621	-3.734727	-1.148943
36	1	0	-2.813305	-1.319541	-1.694898
37	1	0	-2.845895	0.903495	-1.870498
38	6	0	-4.182184	0.030381	0.336912
39	6	0	-3.243496	1.080836	0.473906
40	1	0	-5.083772	0.015821	-0.260090
41	1	0	-3.493944	2.127846	0.355469
42	7	0	-2.642871	-0.652395	1.930046
43	6	0	-1.945807	-1.461707	2.935145
44	1	0	-1.792040	-0.868190	3.839968
45	1	0	-2.568172	-2.328470	3.165360
46	1	0	-0.976944	-1.808225	2.558954
47	6	0	-3.752576	-1.113672	1.161756
48	8	0	-4.224497	-2.270602	1.217937
49	6	0	-2.329253	0.672618	1.615272
50	8	0	-1.439501	1.353259	2.164681
51	6	0	-3.057011	-3.913400	-1.597823
52	1	0	-3.163184	-4.990575	-1.711454
53	1	0	-3.741527	-3.544052	-0.828743
54	1	0	-3.217727	-3.424011	-2.564340
55	29	0	0.671127	-0.309288	-0.255234

exo-TS-(Cu-bipym)

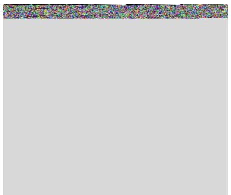

Center Number	Atomic Number	Atomic Type	Coordinates (Angstroms) X	Y	Z
1	6	0	3.949671	-0.545982	-0.514195

2	6	0	5.167245	-1.701932	-2.098031
3	6	0	3.980304	-2.138627	-2.716908
4	1	0	6.144738	-1.987032	-2.472130
5	6	0	2.768621	-1.710289	-2.152872
6	1	0	3.996883	-2.776389	-3.591956
7	1	0	1.811312	-2.003452	-2.567176
8	6	0	3.876063	0.284822	0.723205
9	6	0	4.906499	1.509991	2.391033
10	6	0	3.666063	1.730626	3.019438
11	1	0	5.832446	1.904422	2.796600
12	6	0	2.531526	1.180651	2.405661
13	1	0	3.585441	2.299265	3.937185
14	1	0	1.543415	1.303032	2.832445
15	7	0	5.008991	0.789943	1.247802
16	7	0	2.626054	0.461006	1.257474
17	7	0	5.153287	-0.909035	-0.997905
18	7	0	2.750468	-0.911515	-1.057254
19	7	0	-0.859408	-0.431598	-0.239370
20	6	0	-1.409949	-1.634826	-0.199828
21	6	0	-0.573767	-2.757877	0.288402
22	8	0	0.624173	-2.544322	0.608082
23	6	0	-1.685808	0.653902	-0.657452
24	6	0	-1.094129	1.990880	-0.836524
25	6	0	-0.114918	2.495390	0.056512
26	6	0	-1.555854	2.821734	-1.887629
27	6	0	0.412144	3.779849	-0.116520
28	1	0	0.208121	1.886198	0.895621
29	6	0	-1.017189	4.105568	-2.067138
30	1	0	-2.333874	2.467231	-2.556870
31	6	0	-0.030496	4.587307	-1.186127
32	1	0	1.155409	4.160615	0.577360
33	1	0	-1.371718	4.732224	-2.879240
34	8	0	-1.085571	-4.007324	0.380907
35	6	0	-2.492797	-4.339459	0.042258
36	1	0	-2.574022	-5.400765	0.269082
37	1	0	-3.193274	-3.768208	0.659290
38	1	0	-2.672267	-4.174776	-1.025906
39	1	0	-2.420384	-1.819907	-0.551826
40	1	0	-2.434118	0.381680	-1.406719
41	6	0	-3.288910	-0.317227	1.539512
42	6	0	-3.048838	0.909588	0.885594
43	1	0	-2.712817	-0.785158	2.323742
44	1	0	-2.512745	1.734752	1.338549
45	7	0	-5.113664	0.047209	0.156216
46	6	0	-6.421307	-0.099496	-0.494495
47	1	0	-6.344452	0.096968	-1.567705
48	1	0	-6.766368	-1.122721	-0.328303
49	1	0	-7.139404	0.602971	-0.060532
50	6	0	-4.524725	-0.928223	0.996898
51	8	0	-4.968743	-2.080946	1.192671
52	6	0	-4.311126	1.204727	0.085870
53	8	0	-4.588002	2.230642	-0.555265
54	1	0	0.378875	5.583709	-1.318871
55	29	0	1.133583	-0.410610	0.140603

endo-RC-(Cu-Pt-bipym)

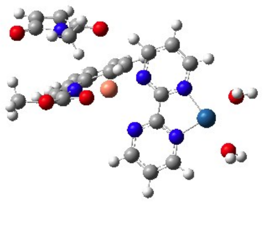

Center Number	Atomic Number	Atomic Type	Coordinates (Angstroms) X	Y	Z
1	6	0	-1.849569	0.271747	0.953490

2	6	0	-3.529823	0.442007	2.590935
3	6	0	-2.533822	0.702653	3.545001
4	1	0	-4.580967	0.404455	2.846359
5	6	0	-1.191234	0.743625	3.117205
6	1	0	-2.798851	0.870906	4.581603
7	1	0	-0.381299	0.949740	3.806785
8	6	0	-1.557451	-0.012891	-0.448650
9	6	0	-2.475962	-0.536562	-2.552249
10	6	0	-1.175078	-0.565254	-3.082134
11	1	0	-3.354574	-0.723503	-3.155312
12	6	0	-0.085296	-0.325402	-2.232208
13	1	0	-1.014153	-0.778402	-4.131258
14	1	0	0.952150	-0.379722	-2.557103
15	7	0	-2.656440	-0.275184	-1.229686
16	7	0	-0.288049	-0.027558	-0.903359
17	7	0	-3.184098	0.222833	1.292083
18	7	0	-0.858836	0.526477	1.815962
19	7	0	3.061515	0.606851	1.160170
20	6	0	3.597501	-0.458248	1.831005
21	6	0	2.828103	-1.704440	1.843097
22	8	0	1.667053	-1.726266	1.305669
23	6	0	3.750642	1.768369	1.141084
24	6	0	3.416665	2.960644	0.415341
25	6	0	2.352740	3.048963	-0.533764
26	6	0	4.223547	4.118447	0.649837
27	6	0	2.102114	4.246799	-1.202090
28	1	0	1.759585	2.167716	-0.762855
29	6	0	3.960250	5.319706	-0.016452
30	1	0	5.048095	4.066240	1.356516
31	6	0	2.898468	5.389551	-0.943048
32	1	0	1.309441	4.306589	-1.941320
33	1	0	4.574846	6.193474	0.171358
34	1	0	2.697518	6.318869	-1.467251
35	8	0	3.294333	-2.830323	2.396485
36	1	0	4.557327	-0.375953	2.334381
37	1	0	4.667557	1.812298	1.735950
38	6	0	6.217148	-0.777002	-1.502313
39	6	0	5.200655	-0.092335	-2.073814
40	1	0	7.254511	-0.495111	-1.384033
41	1	0	5.213432	0.882140	-2.540572
42	7	0	4.309773	-2.145792	-1.422170
43	6	0	3.456550	-3.337730	-1.305872
44	1	0	3.077635	-3.619781	-2.292472
45	1	0	4.074602	-4.151061	-0.919107
46	1	0	2.619569	-3.166787	-0.623120
47	6	0	5.682061	-2.099767	-1.018676
48	8	0	6.243847	-2.976030	-0.350727
49	6	0	3.955106	-0.934568	-2.003066
50	8	0	2.791968	-0.609506	-2.347788
51	6	0	4.672200	-2.992559	2.947786
52	1	0	4.667170	-3.993826	3.374240
53	1	0	5.400080	-2.931667	2.133808
54	1	0	4.858518	-2.252674	3.731280
55	8	0	-5.591776	-0.766618	-1.912602
56	1	0	-6.144913	-0.135232	-2.415731
57	8	0	-6.200006	-0.068186	0.883639
58	1	0	-6.739524	0.748523	0.886285
59	1	0	-5.840013	-1.698991	-2.077800
60	1	0	-6.692702	-0.844272	1.219150
61	29	0	1.217378	0.078987	0.392027
62	78	0	-4.414263	-0.218680	-0.240893

exo-RC-(Cu-Pt-bipym)

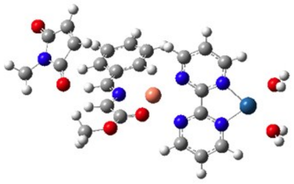

Center Number	Atomic Number	Atomic Type	Coordinates (Angstroms) X	Y	Z
1	6	0	2.275804	-0.267279	-0.806221
2	6	0	3.886333	-0.871870	-2.404607
3	6	0	2.842273	-1.186817	-3.291444
4	1	0	4.928403	-0.980254	-2.676439
5	6	0	1.512416	-1.003840	-2.860422
6	1	0	3.062588	-1.563636	-4.283309
7	1	0	0.669634	-1.228595	-3.503955
8	6	0	2.036028	0.185297	0.554929
9	6	0	3.015754	0.804191	2.600109
10	6	0	1.723902	0.957143	3.141177
11	1	0	3.913158	0.983847	3.176318
12	6	0	0.616087	0.702216	2.320939
13	1	0	1.595808	1.264163	4.172331
14	1	0	-0.398068	0.802140	2.687728
15	7	0	3.159945	0.413147	1.307692
16	7	0	0.771787	0.318500	1.011839
17	7	0	3.598624	-0.413073	-1.155041
18	7	0	1.237981	-0.536198	-1.610627
19	7	0	-2.643054	-0.398845	-0.770595
20	6	0	-3.113038	-1.682063	-0.699313
21	6	0	-2.169156	-2.725785	-0.291877
22	8	0	-0.951697	-2.402042	-0.036764
23	6	0	-3.489257	0.574207	-1.171803
24	6	0	-3.215569	1.980838	-1.272240
25	6	0	-1.988903	2.597282	-0.881047
26	6	0	-4.262494	2.808467	-1.783367
27	6	0	-1.829101	3.978654	-0.985368
28	1	0	-1.180542	1.987549	-0.483868
29	6	0	-4.092913	4.192297	-1.896596
30	1	0	-5.202287	2.355984	-2.086893
31	6	0	-2.878715	4.782827	-1.492482
32	1	0	-0.903163	4.452910	-0.675358
33	1	0	-4.892515	4.809425	-2.290996
34	8	0	-2.527573	-4.002025	-0.181015
35	6	0	-3.908178	-4.511370	-0.474479
36	1	0	-3.863152	-5.555898	-0.174912
37	1	0	-4.641283	-3.959952	0.118166
38	1	0	-4.093954	-4.432232	-1.549833
39	1	0	-4.152401	-1.915983	-0.905692
40	1	0	-4.498833	0.266924	-1.457151
41	6	0	-5.784898	-0.416878	2.342287
42	6	0	-6.449040	0.756804	2.421182
43	1	0	-5.027647	-0.823334	2.998373
44	1	0	-6.357895	1.553674	3.145622
45	7	0	-7.325340	-0.423816	0.591737
46	6	0	-8.242711	-0.859880	-0.473639
47	1	0	-8.251202	-0.141983	-1.297035
48	1	0	-7.915183	-1.838185	-0.831936
49	1	0	-9.258914	-0.947403	-0.079713
50	6	0	-6.295218	-1.181167	1.147982
51	8	0	-5.841642	-2.258031	0.691693
52	6	0	-7.446071	0.814392	1.289869
53	8	0	-8.205762	1.737232	0.976791
54	1	0	-2.747682	5.857472	-1.566674
55	8	0	6.632131	-0.293388	-0.813230
56	1	0	7.145709	-1.121976	-0.725375
57	8	0	6.129131	0.643366	1.935236
58	1	0	6.534136	1.531771	2.004878
59	1	0	7.138174	0.417217	-1.256372
60	1	0	6.566614	-0.016453	2.511274
61	78	0	4.888409	0.087435	0.314525
62	29	0	-0.669024	-0.388345	-0.203802

endo-P-(Cu-Pt-bipym)

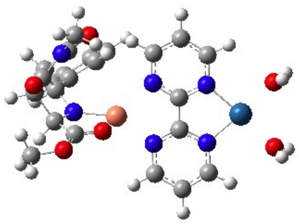

Center Number	Atomic Number	Atomic Type	Coordinates (Angstroms) X	Y	Z
1	6	0	1.706539	-0.192276	-0.966679
2	6	0	3.308091	-0.780815	-2.575052
3	6	0	2.269349	-0.925039	-3.511673
4	1	0	4.346807	-0.944763	-2.830574
5	6	0	0.946609	-0.673843	-3.100386
6	1	0	2.486111	-1.220953	-4.531210
7	1	0	0.108185	-0.762475	-3.779535
8	6	0	1.455669	0.197800	0.416379
9	6	0	2.395009	0.678121	2.508478
10	6	0	1.099907	0.916710	2.996533
11	1	0	3.277094	0.771786	3.128022
12	6	0	-0.000768	0.774953	2.124669
13	1	0	0.950164	1.204219	4.030921
14	1	0	-1.030828	0.938564	2.442258
15	7	0	2.568539	0.313190	1.205846
16	7	0	0.197752	0.408540	0.821063
17	7	0	3.015981	-0.418501	-1.293240
18	7	0	0.674850	-0.299801	-1.816471
19	7	0	-3.090316	0.093971	-1.082256
20	6	0	-3.910610	-1.152499	-1.052777
21	6	0	-2.966074	-2.364735	-0.918427
22	8	0	-1.749420	-2.160914	-0.640522
23	6	0	-4.019590	1.191832	-0.680821
24	6	0	-3.457211	2.598772	-0.713012
25	6	0	-2.398143	3.058546	0.102224
26	6	0	-4.041303	3.496816	-1.634314
27	6	0	-1.945993	4.386728	-0.003053
28	1	0	-1.990416	2.414744	0.870961
29	6	0	-3.596739	4.826834	-1.733121
30	1	0	-4.859982	3.167031	-2.270212
31	6	0	-2.545291	5.276346	-0.914193
32	1	0	-1.156884	4.745764	0.651309
33	1	0	-4.073186	5.504628	-2.433685
34	1	0	-2.213590	6.307953	-0.974108
35	8	0	-3.382367	-3.601729	-1.157665
36	1	0	-4.445632	-1.238320	-2.010838
37	1	0	-4.839111	1.187615	-1.419532
38	6	0	-5.000883	-0.913520	0.091142
39	6	0	-4.680086	0.532382	0.578699
40	1	0	-6.007104	-1.038173	-0.308740
41	1	0	-5.571354	1.085732	0.892306
42	7	0	-4.118860	-1.028031	2.285975
43	6	0	-3.717307	-1.572859	3.596453
44	1	0	-3.696106	-0.764440	4.329827
45	1	0	-4.453879	-2.322675	3.893007
46	1	0	-2.734501	-2.052137	3.539415
47	6	0	-4.823103	-1.787706	1.331961
48	8	0	-5.183078	-2.961180	1.481699
49	6	0	-3.815491	0.257557	1.830436
50	8	0	-2.968602	0.996282	2.374831
51	6	0	-4.785017	-4.004552	-1.491913
52	1	0	-4.690473	-5.042151	-1.805091
53	1	0	-5.391148	-3.922607	-0.588248
54	1	0	-5.163588	-3.393139	-2.317043
55	8	0	5.519815	0.184358	1.936708
56	1	0	6.019195	1.016923	2.061354
57	8	0	6.018367	-0.678656	-0.837899
58	1	0	6.655398	-0.014913	-1.173035
59	1	0	5.843243	-0.539313	2.511197
60	1	0	6.395204	-1.581894	-0.811574
61	29	0	-1.177626	-0.088739	-0.853342

62	78	0	4.291652	-0.148689	0.254575

exo-P-(Cu-Pt-bipym)

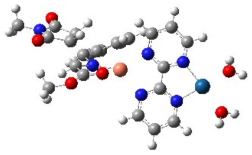

Center Number	Atomic Number	Atomic Type	Coordinates (Angstroms) X	Y	Z
1	6	0	-2.010006	-0.294969	0.743414
2	6	0	-3.460755	-0.769718	2.505119
3	6	0	-2.344574	-1.058379	3.313322
4	1	0	-4.468377	-0.812690	2.889546
5	6	0	-1.057424	-0.915748	2.764093
6	1	0	-2.483271	-1.366317	4.343970
7	1	0	-0.162791	-1.106057	3.343657
8	6	0	-1.882647	0.076729	-0.665692
9	6	0	-2.983657	0.643288	-2.646450
10	6	0	-1.731978	0.756720	-3.277551
11	1	0	-3.901376	0.843916	-3.179086
12	6	0	-0.573942	0.505991	-2.525257
13	1	0	-1.667441	1.033036	-4.323723
14	1	0	0.414271	0.568920	-2.964661
15	7	0	-3.055598	0.287432	-1.331771
16	7	0	-0.659597	0.174007	-1.200289
17	7	0	-3.288789	-0.403256	1.205595
18	7	0	-0.900219	-0.509966	1.464253
19	7	0	2.759212	-0.159256	0.634099
20	6	0	3.567589	-1.407341	0.399739
21	6	0	2.641594	-2.597736	0.245594
22	8	0	1.456746	-2.362597	-0.138191
23	6	0	3.785143	0.921415	0.651957
24	6	0	3.291789	2.341120	0.816247
25	6	0	2.203109	2.852332	0.070303
26	6	0	3.992925	3.206988	1.686134
27	6	0	1.823138	4.199299	0.197545
28	1	0	1.668144	2.207041	-0.629001
29	6	0	3.612219	4.554140	1.816741
30	1	0	4.846659	2.838318	2.249084
31	6	0	2.525906	5.053074	1.074511
32	1	0	1.004032	4.603303	-0.391379
33	1	0	4.162270	5.212819	2.480738
34	8	0	3.023064	-3.844295	0.469383
35	6	0	4.340338	-4.278428	1.053275
36	1	0	4.287854	-5.367367	1.046655
37	1	0	5.152014	-3.938107	0.410429
38	1	0	4.411630	-3.912094	2.074325
39	1	0	4.332588	-1.538616	1.163285
40	1	0	4.560204	0.704212	1.402152
41	6	0	4.330621	-0.946853	-0.995549
42	6	0	4.473732	0.591265	-0.812244
43	1	0	3.676383	-1.298582	-1.796240
44	1	0	3.954456	1.228078	-1.522861
45	7	0	6.621032	-0.443016	-0.839892
46	6	0	8.087422	-0.599964	-0.793629
47	1	0	8.489265	-0.023091	0.039829
48	1	0	8.300961	-1.658953	-0.667774
49	1	0	8.543093	-0.242520	-1.721212
50	6	0	5.746935	-1.503301	-1.068354
51	8	0	6.044707	-2.695168	-1.229978
52	6	0	5.990367	0.823266	-0.785852
53	8	0	6.573420	1.908252	-0.721174
54	1	0	2.236986	6.099226	1.168337
55	8	0	-6.393901	-0.401239	1.027723

56	1	0	-6.614712	-1.267461	1.428097
57	8	0	-6.191250	0.308602	-1.659826
58	1	0	-7.016911	0.796845	-1.437154
59	1	0	-7.108347	0.258133	1.174247
60	1	0	-6.178925	0.004003	-2.591699
61	78	0	-4.711433	-0.034324	-0.193394
62	29	0	0.841775	-0.346136	0.240801

endo-TS-(Cu-Pt-bipym)

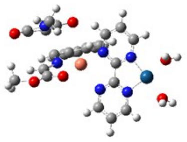

Center Number	Atomic Number	Atomic Type	Coordinates (Angstroms) X	Y	Z
1	6	0	1.779549	-0.183752	-0.953937
2	6	0	3.384164	-0.815098	-2.546414
3	6	0	2.343384	-1.017026	-3.468481
4	1	0	4.423293	-0.980706	-2.799982
5	6	0	1.020243	-0.754758	-3.058663
6	1	0	2.561540	-1.358608	-4.473112
7	1	0	0.178439	-0.878187	-3.728984
8	6	0	1.537372	0.197685	0.435539
9	6	0	2.511549	0.700827	2.513185
10	6	0	1.222778	0.898285	3.036051
11	1	0	3.406657	0.821945	3.108861
12	6	0	0.107487	0.710840	2.200918
13	1	0	1.090757	1.187649	4.071199
14	1	0	-0.923745	0.831117	2.533132
15	7	0	2.659915	0.338912	1.208214
16	7	0	0.279862	0.358533	0.883786
17	7	0	3.096456	-0.398498	-1.281350
18	7	0	0.750768	-0.327927	-1.794280
19	7	0	-3.217336	-0.083647	-0.912552
20	6	0	-3.688385	-1.274297	-1.236964
21	6	0	-2.746458	-2.429164	-1.140090
22	8	0	-1.531253	-2.199149	-0.856290
23	6	0	-4.116320	1.016564	-0.871427
24	6	0	-3.623179	2.406462	-0.953129
25	6	0	-2.415249	2.848593	-0.356120
26	6	0	-4.440449	3.353334	-1.624843
27	6	0	-2.033658	4.192830	-0.441778
28	1	0	-1.814293	2.157277	0.226301
29	6	0	-4.053314	4.699230	-1.712600
30	1	0	-5.374559	3.038573	-2.083379
31	6	0	-2.848722	5.124532	-1.118856
32	1	0	-1.120061	4.531149	0.037797
33	1	0	-4.684616	5.413489	-2.230809
34	1	0	-2.553682	6.167887	-1.174087
35	8	0	-3.151891	-3.679863	-1.352510
36	1	0	-4.711668	-1.425544	-1.572877
37	1	0	-5.045309	0.856587	-1.422859
38	6	0	-5.925105	-0.240098	0.877446
39	6	0	-4.958022	0.793896	0.968273
40	1	0	-6.918852	-0.188856	0.452755
41	1	0	-5.226359	1.840998	1.047810
42	7	0	-4.156791	-1.076837	2.132668
43	6	0	-3.381592	-1.963953	3.009372
44	1	0	-3.181765	-1.459825	3.959495
45	1	0	-3.976402	-2.860894	3.195098
46	1	0	-2.433512	-2.256318	2.544285
47	6	0	-5.380547	-1.467806	1.484858
48	8	0	-5.817564	-2.633155	1.463793
49	6	0	-3.850936	0.247184	1.863042
50	8	0	-2.808724	0.832131	2.245832

51	6	0	-4.578279	-4.109255	-1.528607
52	1	0	-4.509181	-5.191240	-1.618410
53	1	0	-5.157977	-3.836544	-0.642413
54	1	0	-4.986310	-3.680038	-2.447329
55	8	0	5.624716	0.279433	1.914303
56	1	0	6.152486	1.094268	2.038163
57	8	0	6.124378	-0.460301	-0.896916
58	1	0	6.655793	0.257253	-1.298338
59	1	0	5.928312	-0.454883	2.485732
60	1	0	6.612605	-1.307032	-0.848058
61	29	0	-1.208127	-0.142509	-0.404173
62	78	0	4.385113	-0.058764	0.234606

exo-TS-(Cu-Pt-bipym)

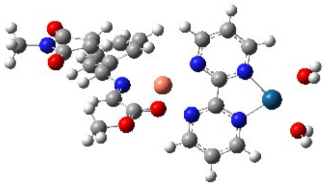

Center Number	Atomic Number	Atomic Type	Coordinates (Angstroms) X	Y	Z
1	6	0	-2.085001	-0.003354	0.774838
2	6	0	-3.574002	-0.354340	2.558993
3	6	0	-2.472907	-0.408923	3.427108
4	1	0	-4.591678	-0.471427	2.907271
5	6	0	-1.178701	-0.231388	2.890728
6	1	0	-2.619995	-0.576693	4.486643
7	1	0	-0.299004	-0.245373	3.522008
8	6	0	-1.942128	0.160812	-0.657492
9	6	0	-3.053917	0.339408	-2.723632
10	6	0	-1.805477	0.440787	-3.355642
11	1	0	-3.986248	0.365191	-3.272152
12	6	0	-0.640144	0.381119	-2.562884
13	1	0	-1.742982	0.553995	-4.430950
14	1	0	0.346998	0.442153	-3.004615
15	7	0	-3.118060	0.198908	-1.370008
16	7	0	-0.712399	0.238623	-1.210602
17	7	0	-3.377760	-0.144627	1.227285
18	7	0	-0.993141	-0.026653	1.559896
19	7	0	2.794738	-0.384770	0.549066
20	6	0	3.114516	-1.646768	0.762694
21	6	0	2.032237	-2.669907	0.679878
22	8	0	0.844704	-2.287789	0.458684
23	6	0	3.857035	0.575956	0.591714
24	6	0	3.545239	2.006878	0.714669
25	6	0	2.456608	2.619999	0.035202
26	6	0	4.417338	2.819426	1.488291
27	6	0	2.244286	3.998038	0.138597
28	1	0	1.809549	2.021008	-0.605190
29	6	0	4.186729	4.196084	1.611433
30	1	0	5.273304	2.374798	1.987308
31	6	0	3.105050	4.791744	0.934290
32	1	0	1.435156	4.473742	-0.406897
33	1	0	4.854987	4.803890	2.211020
34	8	0	2.296441	-3.966767	0.846350
35	6	0	3.657248	-4.554789	1.055035
36	1	0	3.479876	-5.626885	1.012728
37	1	0	4.337217	-4.248664	0.254589
38	1	0	4.029802	-4.274764	2.043503
39	1	0	4.123979	-1.951282	1.029969
40	1	0	4.704236	0.254909	1.204455
41	6	0	4.822043	-0.976490	-1.571710
42	6	0	4.775678	0.382203	-1.194006

43	1	0	4.071176	-1.561062	-2.086339
44	1	0	4.177145	1.118975	-1.718623
45	7	0	6.951525	-0.456301	-0.812657
46	6	0	8.391960	-0.554984	-0.513673
47	1	0	8.578658	-0.575282	0.564270
48	1	0	8.772035	-1.477298	-0.958008
49	1	0	8.903070	0.308098	-0.946191
50	6	0	6.138000	-1.551909	-1.184334
51	8	0	6.457416	-2.756329	-1.164661
52	6	0	6.222192	0.748789	-0.839149
53	8	0	6.662276	1.879363	-0.596619
54	1	0	2.941736	5.862374	1.008534
55	8	0	-6.422270	-0.208862	1.074189
56	1	0	-6.901993	-1.054858	1.180161
57	8	0	-6.135830	0.196993	-1.828999
58	1	0	-6.586156	1.040998	-2.035711
59	1	0	-6.931437	0.556633	1.408706
60	1	0	-6.571370	-0.575288	-2.243951
61	78	0	-4.768781	0.007919	-0.224564
62	29	0	0.780599	-0.139141	0.211219

References.

1 a) C. J. Chang, E. L. Diaz, R. W. Woolfenden, M. D. Grant, *J. Org. Chem.*, 1982, ***47,*** 5318-5321. b) A. L. Webber, S. Masiero, C. Pieraccini, J. Burley, A. S. Tatton, D. Luga, N. T. Pham , P. Spada, P. S. B. Brown, *J. Am. Chem. Soc.*, 2011, **133**, 19777-19795. c) M. Marjaaana, D. K. Klika, A. Hakala, J. Arpalahti, *Inorg. Chem*., 1999, **38,** (3), 571-578.

2 L. Forman, J. C. Fettinger, S. Pieraccini, G. Gottarelli, J. T. Davis, *J. Am. Chem. Soc.,* 2000, **122***,* 4060-4067.

3 S. Yan, C. Zhang, Y. H. Wang, Z. Cao, Z., Zheng, X. P. Hu, *Tetrahedron Letters*, 2013, **54**, 3669-3672.

4 J. F. Bai, L. L. Wang, L. Peng, Y. L. Guo, J. N. Ming, F. Y. Wang, X. Y. Xu, L. X. Wang, *Eur. J. Org. Chem*., 2011, **23**, 4472-4478.
